# A Context-Aware Graph Transformer Framework for microRNA–Gene Regulatory Inference Across Bulk Tumor and Single-Cell Cancer Data

**DOI:** 10.3390/genes17080846

**Published:** 2026-07-23

**Authors:** Jane Ohia, Juan Cui

**Affiliations:** Systems Biology and Biomedical Informatics Laboratory, School of Computing, University of Nebraska-Lincoln, Lincoln, NE 68588, USA; johia2@huskers.unl.edu

**Keywords:** microRNA, non-coding RNA, graph transformer, heterogeneous graph learning, cancer genomics, Bayesian network, single-cell multi-omics, gene regulation, regulatory network inference, tumor heterogeneity

## Abstract

**Background**: MicroRNAs are key post-transcriptional regulators of gene expression and contribute to cancer progression, tumor heterogeneity, and context-dependent regulatory rewiring. However, most computational approaches rely on sequence-based target prediction or bulk expression association and are not designed to jointly model regulatory priors, expression context, and heterogeneous cancer states, particularly when matched single-cell microRNA/mRNA co-profiling data are scarce. **Methods**: We developed a context-aware graph transformer framework for microRNA–gene regulatory analysis across biological resolutions. The framework represents microRNAs, genes, and biological contexts as a heterogeneous graph, where contexts correspond to individual cells in single-cell data and tumor samples or subtype-defined profiles in bulk cohorts. Heterogeneous graph transformer learning generated regulatory embeddings, Bayesian topology optimization refined candidate microRNA–gene interactions, and a dominance-based competition layer with Dominance Share scoring identified master regulators and cooperative target modules. **Results**: We applied miR-CellMap to matched single-cell miRNA/mRNA co-sequencing data from K562 leukemia cells and paired bulk cancer datasets spanning pan-cancer and subtype-specific cohorts, including breast, colon, glioblastoma, lower-grade glioma, and ovarian cancer. The framework identified recurrent and dataset-specific miRNA regulatory programs, including regulators such as miR-186-5p, miR-214-3p, miR-27a-3p, and let-7 family members. Embedding-derived context analysis showed that predicted miRNA target programs were consistently closer to observed context-specific gene programs than random matched gene programs across all seven datasets. Dominance Share analysis further identified cooperative target modules and co-repressed target programs, supporting the use of miR-CellMap for interpretable cancer-focused miRNA regulatory discovery. **Conclusions**: This framework provides an interpretable strategy for mapping conserved, cancer-specific, and context-dependent microRNA–gene regulatory programs across single-cell and bulk cancer datasets.

## 1. Introduction

MicroRNAs (miRNAs) are small non-coding RNAs that regulate gene expression after transcription by guiding RNA-induced silencing complexes to target transcripts, leading to mRNA degradation, translational repression, or both [1,2,3,4,5]. Although miRNAs do not encode proteins, they exert broad regulatory effects because a single miRNA can target many transcripts, and a single transcript can be regulated by multiple miRNAs. This many-to-many regulatory structure allows miRNAs to shape developmental programs, immune responses, stress adaptation, differentiation, and cellular homeostasis [1,5,6]. In cancer, dysregulated miRNA activity has been associated with oncogenic signaling, tumor-suppressive programs, metastatic progression, immune escape, therapy response, and tumor heterogeneity, making miRNA-mediated regulation an important layer of cancer biology [7,8,9,10].

A central challenge in studying miRNA regulation is that miRNA–gene interactions are highly context-dependent. A predicted miRNA binding site does not necessarily indicate an active regulatory interaction in a specific tumor type, cell state, or microenvironmental condition. Conversely, an interaction that appears weak in one biological setting may become functionally important in another. This context dependency reflects variation in miRNA abundance, target transcript expression, RNA-binding proteins, transcript isoforms, competing endogenous RNAs, tumor composition, and cell-state-specific regulatory programs [11,12,13]. Therefore, miRNA regulatory analysis cannot be reduced to static target lookup alone. It requires computational strategies that can connect prior biological knowledge with expression-derived evidence and the biological context in which regulation occurs.

Existing miRNA–gene interaction methods provide complementary but incomplete views of this problem. Sequence-based target prediction tools prioritize candidate targets using seed matching, site conservation, site accessibility, and transcript sequence features [14]. Curated resources and experimental databases, including miRTarBase and ENCORI/starBase [15,16,17], provide valuable collections of validated or evidence-supported miRNA–target interactions. Expression-based methods use correlation, anti-correlation, regression, or network inference to identify interactions supported by matched molecular profiles [18,19]. However, these strategies have important limitations. Sequence-based prediction may generate large candidate sets that are not active in each biological context, while expression anti-correlation may miss translational repression, indirect regulation, or interactions masked by tumor mixture, shared upstream regulators, or nonlinear regulatory effects. These limitations have produced diverging assumptions in the field: some approaches emphasize conserved sequence evidence as the most reliable starting point, whereas others prioritize context-specific expression associations. In practice, both views capture useful information, but neither alone is sufficient for context-aware miRNA regulatory inference.

Single-cell and multi-omic technologies have expanded the ability to study regulatory heterogeneity at cellular resolution. Single-cell RNA sequencing has transformed cancer genomics by revealing rare cell populations, transitional states, and tumor ecosystem organization that are obscured in bulk measurements [20,21]. Multi-omic methods further enable multiple molecular layers to be measured in the same cell or sample, providing a richer view of how regulatory state, transcriptional activity, and cellular identity are coupled [22,23,24,25,26]. Emerging single-cell miRNA/mRNA co-profiling approaches are particularly important because they create opportunities to examine post-transcriptional regulation directly within matched cellular contexts [13,27,28]. However, such datasets remain scarce in cancer. As a result, a practical miRNA regulatory framework should be able to operate across biological resolutions, using single-cell co-profiling data when available and paired bulk tumor cohorts when single-cell miRNA measurements are limited.

These data structures introduce major computational challenges. Single-cell and cancer multi-omic datasets are high-dimensional, sparse, noisy, and heterogeneous, often involving multiple entity types such as cells, tumor samples, genes, miRNAs, cancer subtypes, and curated regulatory relationships [20,22,23,29]. Conventional matrix-based analyses remain useful for normalization, clustering, differential expression, and visualization, but they are less suited for representing multi-type regulatory systems in which biological entities have different roles and relationships. Graph-based representation learning provides a natural alternative because molecular and biological entities can be represented as nodes, while their regulatory, expression-derived, or prior-supported relationships can be represented as edges [30,31,32]. This structure allows computational models to learn from both node features and network topology.

Heterogeneous graph learning is especially relevant for miRNA regulatory modeling because miRNAs, genes, and biological contexts are not interchangeable entities. They have different biological meanings and participate in different types of relationships. Heterogeneous graph transformers extend graph neural networks by allowing multiple node and edge types to be modeled within a unified attention-based architecture [33]. In a miRNA–gene regulatory setting, miRNAs can be represented as regulatory nodes, genes as target nodes, and cells, tumor samples, or subtype-defined profiles as biological context nodes. Prior-supported miRNA–gene relationships can then be integrated with expression-derived structure, allowing candidate regulatory interactions to be scored in a context-aware manner. This approach moves beyond treating miRNA targeting as a fixed database lookup and instead places prior evidence, expression structure, and biological context into the same computational space.

However, representation learning alone does not fully resolve regulatory inference. Embeddings can prioritize candidate interactions, but the resulting networks may still contain redundant, weakly supported, or locally ambiguous edges. Bayesian network modeling offers a complementary strategy because it can refine candidate topology using probabilistic dependency structure and conditional relationships among variables [34,35]. When combined with graph-transformer-derived regulatory scores, Bayesian topology optimization can reduce the candidate network while preserving biologically supported structure. An additional interpretability problem remains: many miRNAs may target the same gene, and their regulatory effects may be competitive, cooperative, or context specific. Therefore, a gene-centered dominance framework is needed to quantify the relative contribution of each miRNA within a local regulatory neighborhood and to identify master regulators and cooperative target modules.

In this study, we introduce miR-CellMap, a context-aware graph transformer framework for mapping miRNA–gene regulatory landscapes across single-cell and bulk cancer datasets. By integrating regulatory priors, expression-derived context, heterogeneous graph transformer learning, Bayesian topology optimization, and dominance-based competition analysis, the framework moves beyond static target prediction and expression association alone. The dominance-based competition layer quantifies local regulatory hierarchy, enabling the identification of master regulators and cooperative target modules. We apply miR-CellMap to matched single-cell miRNA/mRNA co-sequencing data from K562 leukemia cells and paired bulk cancer datasets spanning pan-cancer and cancer subtype cohorts, including breast, colon, glioblastoma, lower-grade glioma, and ovarian cancer. The main aim of this work is to determine whether a unified graph-based framework can recover evidence-supported miRNA–gene interactions while also identifying interpretable regulatory programs across biological contexts. The results support this aim, showing that the Bayesian-optimized network achieves strong performance under a high-confidence negative evaluation setting and that the refined dominance network highlights recurrent and dataset-specific miRNA regulatory programs relevant to cancer.

This study contributes a cancer-focused, context-aware framework for miRNA–gene regulatory discovery. Unlike conventional miRNA target prediction tools that primarily rely on sequence features or curated interaction evidence, miR-CellMap integrates paired miRNA/mRNA expression, regulatory priors, and biological context to infer cancer-associated regulatory programs [15,17,36,37]. Unlike generic GNN/HGT biological graph models, miR-CellMap explicitly models a heterogeneous miRNA–gene–context graph, where contexts represent individual cells or tumor samples [30,33]. The Bayesian step is used as a post-HGT topology optimizer to refine candidate regulatory edges, while Dominance Share provides a target-normalized ranking strategy for identifying competing miRNAs, master regulators, and cooperative target modules. Together, these components define miR-CellMap as an interpretable framework for cancer-focused miRNA regulatory discovery rather than a standalone target prediction tool.

## 2. Materials and Methods

### 2.1. Study Design and Workflow Overview

**Figure 1:**
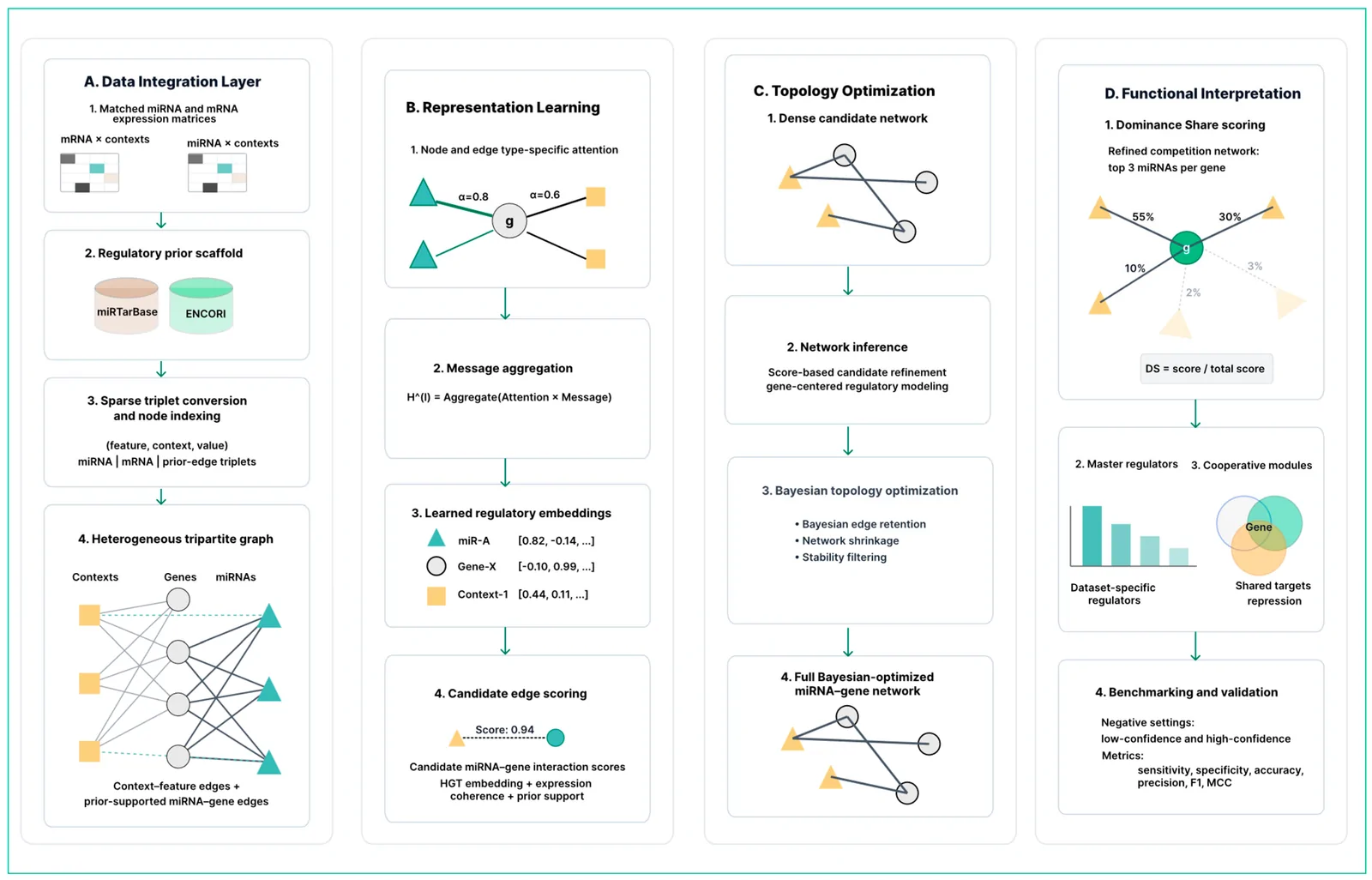
Overview of the miR-CellMap workflow. The framework integrates matched microRNA and mRNA expression profiles with curated regulatory prior evidence, converts aligned expression matrices into sparse triplet format, constructs a heterogeneous tripartite graph linking contexts, microRNAs, and genes, learns regulatory embeddings using heterogeneous graph transformer learning, refines candidate interactions through network inference and Bayesian topology optimization, and performs Dominance Share-based regulatory interpretation and validation.

This study developed and evaluated miR-CellMap, a context-aware computational framework for inferring microRNA–gene regulatory networks across single-cell and bulk cancer datasets. The framework (Figure 1) was designed to integrate matched microRNA and mRNA expression profiles with curated regulatory prior knowledge, while preserving the biological context in which each regulatory interaction is observed. In single-cell data, context was defined at the level of individual cells, whereas in bulk tumor cohorts, context was represented by tumor samples or subtype-defined profiles. This design reflects the increasing need for regulatory inference methods that can operate across heterogeneous molecular datasets and biological resolutions [20,22,23,29]. The miR-CellMap workflow consisted of expression preprocessing and feature alignment, sparse triplet conversion, regulatory prior integration, heterogeneous tripartite graph construction, heterogeneous graph transformer learning, network inference, Bayesian topology optimization, dominance-based regulatory competition analysis, and downstream benchmarking. Graph transformer learning was used to generate regulatory embeddings from the heterogeneous microRNA–gene–context graph (Figure 1), building on graph representation learning approaches for multi-type biological systems [30,31,33]. The resulting candidate network was refined using Bayesian topology optimization, followed by Dominance Share-based analysis to identify master regulators and cooperative target modules. This workflow enabled both quantitative evaluation of inferred regulatory interactions and interpretable analysis of context-dependent microRNA regulatory programs. By sequentially integrating prior-supported edges with expression-derived graph structure, miR-CellMap bridges the gap between static regulatory knowledge and the dynamic regulatory landscapes observed in complex cancer states.

### 2.2. Data Sources and Cancer Cohorts

We applied miR-CellMap to datasets representing two biological resolutions: matched single-cell miRNA/mRNA co-profiling data and paired bulk tumor multi-omic cancer cohorts. The single-cell dataset was obtained from GSE114071, which contains small RNA sequencing and RNA sequencing profiles generated from K562 leukemia cells using a half-cell co-sequencing strategy [38]. This dataset was included to examine whether the framework could model miRNA–gene regulatory structure when miRNA and mRNA measurements were available within matched single-cell contexts.

For bulk cancer analysis, we used paired miRNA and mRNA profiles from MLOmics, a cancer multi-omic resource constructed to support machine-learning and bioinformatics analyses across tumor cohorts [39]. The selected bulk datasets included one pan-cancer cohort and five gold-standard cancer subtype cohorts: breast cancer (GS-BRCA), colon adenocarcinoma (GS-COAD), glioblastoma (GS-GBM), lower-grade glioma (GS-LGG), and ovarian cancer (GS-OV). In these datasets, biological context was represented by tumor samples or subtype-defined profiles rather than individual cells.

In addition to expression profiles, curated miRNA–gene regulatory evidence was obtained from miRTarBase and ENCORI/starBase [15,16,17]. These resources provided experimentally validated and/or multi-source evidence-supported miRNA–gene interactions used to construct the regulatory prior scaffold and define reference-positive interaction sets for benchmarking.

All datasets and databases used in this study were publicly available and de-identified. No new biological specimens, laboratory materials, laboratory equipment, or physical devices were used in this computational study. All input materials were publicly available datasets and databases, including GEO accession GSE114071, MLOmics, miRTarBase, and ENCORI/starBase. Computational analyses were performed using high-performance computing resources provided by the University of Nebraska–Lincoln Holland Computing Center, Lincoln, NE, USA. Dataset-level characteristics, including the number of retained contexts, miRNAs, genes, candidate regulatory edges, and final inferred network edges after preprocessing and model construction, are reported in the Section 3.

### 2.3. Preprocessing, Normalization, and Feature Alignment

All datasets were processed independently to preserve their original biological resolution while ensuring that matched miRNA and mRNA matrices were aligned within each dataset. For the pan-cancer cohort, numeric cancer labels were first mapped to TCGA cancer-type annotations using the accompanying clinical records from the MLOmics resource [39]. Priority cancer types were then selected, and matched mRNA, miRNA, numeric label, clinical, and sample-mapping files were extracted in a consistent sample order. For the gold-standard subtype cohorts, mRNA, miRNA, and subtype label files were validated across the available MLOmics feature scales, with final downstream analyses performed using the prepared subtype-specific feature matrices [39]. Source-provided processed or normalized expression values were used for downstream analysis, and datasets were not globally re-normalized across cohorts because all model construction and evaluation steps were performed within each dataset-specific context.

For the single-cell K562 dataset, miRNA and mRNA matrices were aligned by extracting shared half-cell identifiers from the corresponding small RNA sequencing and RNA sequencing files [38]. Only one-to-one matched cells present in both modalities were retained, and the aligned feature-by-cell matrices were written with standardized cell identifiers. This ensured that each miRNA profile and mRNA profile represented the same biological context before graph construction.

After alignment and identifier normalization, each dataset was audited against the regulatory prior feature space to confirm overlap between retained miRNAs, genes, and evidence-supported miRNA–gene pairs from miRTarBase and ENCORI/starBase [15,16,17]. These preprocessing steps produced matched and identifier-compatible miRNA and mRNA matrices for sparse triplet conversion, heterogeneous graph construction, and downstream network inference.

### 2.4. Regulatory Prior Construction and miRNA–Gene Evidence Integration

Curated miRNA–gene regulatory priors were constructed to define a biologically grounded candidate space for downstream graph construction and network inference. Regulatory evidence was obtained from ENCORI/starBase and miRTarBase, two complementary resources that provide CLIP-supported binding evidence and experimentally validated miRNA–target interactions, respectively [15,16,17]. After filtering, 293,936 unique ENCORI miRNA–gene pairs were retained when the target gene was annotated as protein-coding and at least one CLIP experiment supported the interaction. In addition, 8667 unique miRTarBase miRNA–gene pairs were retained when both the miRNA and target gene were human, the interaction was annotated as a functional miRNA–target interaction, and the evidence included at least one strong experimental assay, such as a luciferase reporter assay, qRT-PCR, or Western blot. After merging and deduplication, the final master regulatory prior contained 299,848 unique miRNA–gene pairs.

For downstream analyses, each retained prior edge was annotated with its source database, evidence type, experimental support, mature-arm status, and source record count. Edges supported by both ENCORI/starBase and miRTarBase were preserved as dual-source evidence-supported interactions and were later used to define high-confidence reference-positive sets for benchmarking. This prior integration strategy allowed miR-CellMap to begin with a biologically informed regulatory scaffold while still permitting downstream graph learning and Bayesian topology optimization to prioritize context-specific regulatory interactions.

### 2.5. Sparse Triplet Conversion and Unified Matrix Formatting

To support scalable graph construction from high-dimensional expression matrices, each aligned mRNA and miRNA matrix was converted into a sparse triplet format. This step preserved only observed non-zero expression values and removed missing entries, reducing storage requirements while retaining the expression relationships needed to construct context–feature edges. Sparse representations are commonly used in single-cell and multi-omic analysis because molecular matrices are often high-dimensional and contain many zero-valued entries [20,22,29].

For each dataset, mRNA and miRNA matrices were first checked to ensure that their context columns were identical and in the same order. Let $C_{d}^{mRNA}$ and $C_{d}^{miRNA}$ denote the ordered context sets for the mRNA and miRNA matrices in dataset d. Dataset (d) was retained for triplet conversion only if:

$$C_{d}^{mRNA}=C_{d}^{miRNA}$$

For an expression matrix $X_{d},\;\mathrm{where}\;x_{fc}$ represents the expression value of feature (f) in context (c), the sparse triplet representation was defined as:

$$T\left(X_{d}\right)=\left\{\left(f,c,x_{fc}\right)|x_{fc}\neq 0,x_{fc}\;\mathrm{is}\;\mathrm{not}\;\mathrm{missing}\right\}$$

Separate sparse triplet files were generated for mRNA and miRNA expression values, with each record storing the feature identifier, context identifier, and expression value. In parallel, dataset-specific gene ID, miRNA ID, and observation ID files were written to preserve a unified indexing system for downstream heterogeneous graph construction. This formatting step provided a compact and consistent representation of context–gene and context–miRNA relationships across all single-cell and bulk cancer datasets.

### 2.6. Heterogeneous Tripartite Graph Construction

For each dataset, the aligned expression triplets and dataset-specific regulatory priors were used to construct a heterogeneous tripartite graph linking biological contexts, genes, and miRNAs. Graph-based representations are well suited for multi-type biological systems because they preserve both molecular features and relational structure among different entity classes [30,31,32]. In this study, biological contexts corresponded to cells in the single-cell dataset and to tumor samples or subtype-defined profiles in the bulk cancer datasets.

For dataset d, the graph was defined as:

$$G_{d}=\left(V_{d},E_{d},\tau ,\rho \right)$$

where $\left(V_{d}\right)$ is the node set, $\left(E_{d}\right)$ is the edge set, τ maps each node to a node type, and $\left(\rho \right)$ maps each edge to a relation type. The node set contained three node classes:

$$V_{d}=C_{d}\cup G_{d}\cup M_{d}$$

where $\left(C_{d}\right)$ denotes contexts, $\left(G_{d}\right)$ denotes genes, and $\left(M_{d}\right)$ denotes miRNAs. The edge set combined expression-derived and prior-supported regulatory relationships:

$$E_{d}=E_{C,G}\cup E_{C,M}\cup E_{M,G}$$

Here $E_{C,G}\;\mathrm{represented}\;\mathrm{context}-\mathrm{gene}\;\mathrm{expression}\;\mathrm{edges},\;\left(E_{C},M\right)$ represented context–miRNA expression edges, and $\left(E_{M},G\right)\;\mathrm{represented}\;\mathrm{prior}-\mathrm{supported}\;\mathrm{miRNA}-\mathrm{gene}\;\mathrm{regulatory}\;\mathrm{edges}.\;\mathrm{For}\;\mathrm{the}\;\mathrm{context}-\mathrm{featuree}\;\mathrm{dges}\left(E_{C},G\right)\;\mathrm{and}\;\left(E_{C,M}\right)$, edge weights were defined by the normalized expression intensity from the sparse triplets, allowing expression magnitude to be preserved during graph construction and reconstruction-based representation learning. miRNA–gene prior edges were annotated with evidence attributes, including match type, ENCORI support, miRTarBase support, mature-arm status, CLIP/degradome evidence counts, and source record count. Reverse edges were also added for each relation type to support bidirectional message passing during heterogeneous graph learning. Each dataset-specific graph was saved as a PyTorch Geometric heterogeneous graph object, with associated metadata describing node counts, edge counts, edge types, and prior-edge attributes [40].

### 2.7. Heterogeneous Graph Transformer Representation Learning

Regulatory representation learning was performed using a heterogeneous graph transformer (HGT) autoencoder. HGT models are designed for graphs containing multiple node and edge types, making them appropriate for learning from context, gene, and miRNA nodes within a shared regulatory graph [33]. For each dataset-specific graph, gene and miRNA node features were reconstructed from the sparse expression triplets as feature-by-context matrices, while context nodes were initialized using an identity feature representation. This identity-based context representation allowed each cell or tumor sample to retain a unique context-specific encoding when no additional external context features were available. These node features were projected into a shared hidden space before heterogeneous message passing.

For a node $\left(v\right)\;\mathrm{with}\;\mathrm{node}\;\mathrm{type}\;\left(\tau \left(v\right)\right),$ the initial hidden representation was computed as:

$$h_{v}^{\left(0\right)}=\mathrm{ReLU}\left(W_{\tau \left(v\right)}x_{v}\right)$$

where $\left(x_{v}\right)$ is the input feature vector and $\left(W_{\tau \left(v\right)}\right)$ is a node-type-specific linear transformation. HGT message passing was then applied across the heterogeneous graph:

$$h_{v}^{\left(l+1\right)}={HGT}^{\left(l\right)}\left(h_{v}^{\left(l\right)},N\left(v\right),\rho \right)$$

where $\left(N\left(v\right)\right)$ denotes the typed neighborhood of node $\left(v\right)$ and *ρ* denotes the relation type associated with each edge. The model used two HGT layers, 128 hidden channels, four attention heads, and was trained for 100 epochs using the Adam optimizer with a learning rate of 0.001.

The HGT encoder was trained in an autoencoder framework to reconstruct observed context–gene and context–miRNA expression edges. Predicted expression values were obtained by concatenating the learned embeddings of the connected node pairs and passing them through modality-specific decoders:

$$x\;{ˆ}_{cg}=f_{G}\left(\left[z_{c}∥z_{g}\right]\right),\;x\;{ˆ}_{cm}=f_{M}\left(\left[z_{c}∥z_{m}\right]\right)$$

where $\left(z_{c}\right),\left(z_{g}\right),\;\mathrm{and}\;\left(z_{m}\right)$ are the learned embeddings of context (c), gene (g), and miRNA (g), respectively. The training objective minimized the combined reconstruction error for gene and miRNA expression edges:

$$L=\frac{1}{\left|E_{C,G}\right|}\sum_{\left(c,g\right)\in E_{C,G}}{\left(x\;{ˆ}_{cg}-x_{cg}\right)}^{2}+\frac{1}{\left|E_{C,M}\right|}\sum_{\left(c,m\right)\in E_{C,M}}{\left(x\;{ˆ}_{cm}-x_{cm}\right)}^{2}$$

After training, the final embeddings were exported for contexts, genes, and miRNAs. For each prior-supported miRNA–gene edge, an HGT interaction score was computed from the learned miRNA and gene embeddings:

$$s_{mg}^{HGT}=\sigma \left(z_{m}^{T}z_{g}\right)$$

where σ is the sigmoid function. These HGT-derived interaction scores were used as graph-based regulatory evidence for downstream network inference and Bayesian topology optimization.

Because HGT training reconstructs context–feature expression relationships rather than experimentally measured miRNA perturbation effects, the resulting miRNA–gene scores represent embedding-derived regulatory plausibility. These scores were therefore integrated with prior evidence and expression coherence before Bayesian refinement.

### 2.8. Network Inference and Bayesian Topology Optimization

Network inference was performed by integrating graph-derived regulatory evidence, expression coherence, and prior support for each candidate miRNA–gene interaction. Candidate edges were obtained from prior-supported miRNA–gene pairs scored by the heterogeneous graph transformer. For each miRNA m and target gene g, expression coherence was computed using the aligned expression profiles across contexts, including Pearson correlation and cosine-based expression similarity. This allowed the inferred network to retain both embedding-based regulatory evidence and dataset-specific expression structure.

For each candidate edge, a hybrid regulatory score was computed as:

$$S_{\left\{mg\right\}}=\alpha H_{\left\{mg\right\}}+\beta EC_{\left\{mg\right\}}+\gamma P_{\left\{mg\right\}}$$

where $H_{\left\{mg\right\}}$ is the HGT interaction score, $EC_{\left\{mg\right\}}$ is the expression-coherence score, $P_{\left\{mg\right\}}\;$ is the prior-strength score, and α, β, and γ are weighting parameters. To account for multiple miRNAs competing for the same target gene, each score was normalized within its target-gene neighborhood:

$$W_{mg}=\frac{S_{mg}}{\sum_{m^{\prime }\in R_{g}}S_{m^{\prime }g}}$$

where $R_{g}$ is the set of candidate miRNA regulators for gene g. The pre-Bayesian regulatory score was then defined as:

$$S_{mg}^{preBN}=S_{mg}\times W_{mg}$$

Bayesian topology optimization was then applied separately within each target-gene subnetwork. For each target gene, candidate miRNA regulators were discretized into quantile-based expression states, and score-based Bayesian structure learning was performed using a Bayesian Dirichlet equivalent uniform score and hill-climbing search [34,41,42]. The optimization constrained candidate structures so that retained directed edges represented miRNA-to-target-gene regulatory relationships. The selected post-Bayesian network was rescored using a second target-level competition normalization:

$$S_{mg}^{\mathrm{final}}=S_{mg}\times W_{mg}^{\mathrm{postBN}}$$

where $W_{mg}^{\mathrm{postBN}}$ is the normalized competition weight among Bayesian-retained regulators for gene g. The resulting full Bayesian-optimized network was used as the primary inferred miRNA–gene regulatory network for each dataset.

### 2.9. Dominance Share-Based Competition Analysis and Refined Network Construction

To improve interpretability of the Bayesian-optimized network, we quantified miRNA regulatory dominance at the target-gene level. This step was designed to identify the strongest miRNA regulators for each target while preserving cooperative regulatory structure. For each retained miRNA–gene edge, Dominance Share was calculated as the proportion of the target gene’s total regulatory score contributed by that miRNA:

$$DS_{mg}=\frac{S_{mg}^{\mathrm{final}}}{\sum_{m^{\prime }\in R_{g}}S_{m^{\prime }g}^{\mathrm{final}}}$$

where $DS_{mg}$ represents the Dominance Share of miRNA m for target gene g, and R_g is the set of Bayesian-retained miRNA regulators for gene g. Edges were ranked within each target gene by final score and Dominance Share. The refined competition network was then constructed by retaining the top k miRNA regulators per target gene:

$$E_{\mathrm{refined}}=\left\{\left(m,g\right)\in E_{\mathrm{BN}}:\mathrm{ran}k_{g}\left(m\right)\leq k\right\}$$

where $E_{\mathrm{BN}}$ is the full Bayesian-optimized network and k = 3 in this study. This retained the strongest local regulators for each target while reducing dense candidate neighborhoods.

Master regulator scores were computed by aggregating final regulatory scores across all retained targets for each miRNA:

$$MR_{m}=\sum_{g\in T_{m}}S_{mg}^{\mathrm{final}}$$

where $T_{m}$ is the set of target genes regulated by miRNA m. In parallel, cooperative target modules were identified by detecting genes regulated by multiple retained miRNAs, with emphasis on genes regulated by at least three miRNAs and genes with multiple negatively correlated miRNA regulators. This dominance-based refinement produced an interpretable network for master regulator ranking, target-level competition analysis, and cooperative module detection.

### 2.10. Context-Aware Regulatory Module Detection and Advanced Regulatory Modeling

Context-aware regulatory module detection was performed to identify miRNA–gene regulatory patterns enriched within specific biological contexts. Contexts represented individual cells in the single-cell dataset and tumor samples or subtype-defined profiles in the bulk datasets. For each dataset, context-associated miRNA programs and marker genes were used to select a representative regulatory context. The selected context was defined as the context with the largest aggregate program-separation score:

$$C^{=\backslash }operatornameargmaxC\sum m\in M_{C}\Delta_{mC}$$

where $\Delta_{mC}$ represents the separation between random and observed miRNA program distances for miRNA m in context C. Within the selected context, candidate regulatory modules were constructed by intersecting context-associated miRNAs, context marker genes, and the final Bayesian-optimized regulatory network.

For each selected context, context-specific regulator scores were calculated by summing final miR-CellMap scores across retained target genes:

$$CSR_{mC}=\sum_{g\in T_{mC}}S_{mg}^{\mathrm{final}}$$

where $CSR_{mC}$ is the context-specific regulator score for miRNA m in context C, and $T_{mC}$ is the set of context-associated target genes regulated by m. The highest-ranking context-specific miRNAs and their top predicted targets were used to generate context-aware regulatory modules. A correlation-ranked comparison module of matched size was also generated to distinguish graph/Bayesian regulatory structure from expression correlation alone.

Advanced regulatory modeling was then used to compare regulatory programs across datasets. Top context-specific regulators were ranked within each dataset and converted into rank-normalized scores:

$$R_{m,d}=1-\frac{{\mathrm{rank}}_{m,d}-1}{K}$$

where $R_{m,d}$ is the rank-based regulator strength for miRNA m in dataset d, and K is the number of top regulators considered. Recurrent regulators, dataset-specific regulators, and cross-dataset module similarities were then identified. Module similarity between datasets was quantified using Jaccard overlap of regulator sets, target sets, and inferred edge sets:

$$J\left(A,B\right)=\frac{\left|A\cap B\right|}{\left|A\cup B\right|}$$

These analyses provided an interpretable view of conserved, cancer-specific, and single-cell-to-bulk regulatory patterns across miR-CellMap outputs.

### 2.11. Benchmarking and Functional Validation

Benchmarking was performed to evaluate whether inferred miRNA–gene interactions recovered evidence-supported regulatory relationships more effectively than baseline approaches. Dual-source evidence-supported interactions were defined as miRNA–gene pairs supported by both ENCORI/starBase and miRTarBase after identifier harmonization and restriction to the dataset-specific candidate space. These interactions were used for conservative recovery analysis of previously reported miRNA–gene relationships, not as an independent ground-truth validation set, because ENCORI/starBase and miRTarBase also contributed to construction of the regulatory prior scaffold [15,16,17]. Evaluation was performed using both exact miRNA matching and stem-level matching, where mature-arm suffixes were removed to assess broader miRNA-family-level recovery.

For each dataset, recovery was assessed within the modeled candidate space by comparing inferred miRNA–gene edges with the dual-source evidence-supported interaction set. The primary recovery summary was defined as:

$$R_{d}=\frac{\left|E_{d}^{pred}\cap E_{d}^{dual}\right|}{\left|E_{d}^{dual}\right|}$$

where $E_{d}^{pred}$ denotes the inferred miRNA–gene edge set for dataset d, and $E_{d}^{dual}$ denotes the dual-source evidence-supported miRNA–gene set for the same dataset. Additional low- and high-confidence background sets were used only for conservative sensitivity analysis. These background sets were not treated as experimentally confirmed negative interactions because unreported or single-source-supported miRNA–gene pairs may still represent biologically valid interactions.

The full Bayesian network and refined top-3 competition network were evaluated against these reference sets. Three matched-size baseline methods were also included: correlation-ranked edges, HGT-score-ranked edges, and prior-support-ranked edges. Each baseline was restricted to the same number of predicted edges as the refined competition network to support fair comparison.

Binary classification performance was summarized using sensitivity, specificity, accuracy, precision, F1 score, and Matthew’s correlation coefficient [43,44]. These metrics were defined as:

$$\mathrm{Sensitivity}=\frac{TP}{TP+FN}$$

$$\mathrm{Specificity}=\frac{TN}{TN+FP}$$

$$\mathrm{Precision}=\frac{TP}{TP+FP}$$

$$F1=\frac{2\times \mathrm{Precision}\times \mathrm{Sensitivity}}{\mathrm{Precision}+\mathrm{Sensitivity}}$$

$$\mathrm{MCC}=\frac{TP\times TN-FP\times FN}{\sqrt{\left(TP+FP\right)\left(TP+FN\right)\left(TN+FP\right)\left(TN+FN\right)}}$$

In addition to binary benchmarking, functional validation tables were created for top-ranked master regulators, literature-supported pathways, dominance-based regulator statistics, and cooperative target modules. This combined evaluation strategy assessed both predictive recovery of evidence-supported miRNA–gene interactions and biological interpretability of the inferred regulatory networks.

To provide an independent benchmark using experimentally verified interactions, we additionally evaluated miR-CellMap on an A549 single-cell miRNA–mRNA co-profiling dataset previously analyzed in our recent miR-CellMap study [45]. The dataset, preprocessing, and experimentally supported reference interactions have been described in detail previously [45] and are used here solely for benchmarking the graph transformer framework.

### 2.12. Software Implementation

The miR-CellMap workflow was implemented as a reproducible Python-based computational pipeline using Python version 3.10.20. The pipeline was organized into sequential scripts for data preprocessing, prior construction, sparse triplet conversion, heterogeneous graph construction, HGT autoencoder training, Bayesian topology optimization, dominance-based network refinement, context-aware module detection, advanced regulatory modeling, and benchmarking. All analyses were performed independently for each dataset to preserve dataset-specific context and avoid cross-cohort normalization artifacts.

Core data processing and numerical operations were performed using pandas version 2.3.3, NumPy version 2.2.6, and SciPy version 1.15.2 [46,47,48]. Heterogeneous graph construction and graph transformer learning were implemented using PyTorch version 2.5.1 and PyTorch Geometric version 2.7.0 [40,49]. Bayesian topology optimization was performed using score-based structure learning implemented through pgmpy version 1.1.2. PCA-based embedding visualization and related machine-learning utilities were performed using scikit-learn version 1.7.2. UMAP-based context visualization was performed using UMAP-learn version 0.5.12 [50]. Network-level summaries and visualizations were generated using NetworkX version 3.4.2 and Matplotlib version 3.10.9 [51,52]. Intermediate files, including sparse triplets, graph objects, HGT embeddings, Bayesian-optimized networks, refined dominance networks, benchmark tables, and figure-ready outputs, were written to structured dataset-specific output directories.

## 3. Results

### 3.1. Methodological Evaluation and Benchmark Performance

#### 3.1.1. Data Summary and Prior Evidence Coverage

The heterogeneous graphs constructed by miR-CellMap comprised substantial dataset-specific node and interaction spaces across both bulk and single-cell settings (Table 1). Across the seven datasets, the framework modeled 5103 biological context nodes, 51,501 gene nodes, and 4949 miRNA nodes. Candidate regulatory interactions retained from the integrated prior knowledge database ranged from 15,271 interactions in the pan-cancer cohort to 275,658 interactions in the K562 dataset. Following Bayesian topology optimization, the final regulatory networks contained between 10,369 interactions (OV) and 99,506 interactions (K562). Collectively, the datasets included 5084 bulk tumor samples and 19 matched single-cell half-cell profiles, providing diverse biological contexts for regulatory network inference and benchmarking.

#### 3.1.2. A549 Benchmarking and Evidence-Recovery Evaluation Across Cancer Datasets

miR-CellMap was assessed under two complementary evaluation settings. First, we performed a ground-truth benchmark using an independently curated A549 single-cell miRNA–mRNA co-profiling dataset from our recently developed miR-CellMap framework [45], where experimentally supported miRNA–gene interactions were available for direct comparison. The dataset, preprocessing procedures, and reference interaction set have been described previously [45]. Within this benchmark, the refined network recovered 220 of 255 experimentally supported interactions (86.27% sensitivity). Under the high-confidence negative setting, the refined network achieved 99.92% exact-match accuracy, 100.00% specificity, 92.63% F1 score, and an MCC greater than 0.928, demonstrating robust recovery of experimentally supported regulatory interactions (Table 2).

The second setting was discovery-oriented evaluation in cancer cohorts, where independent held-out ground-truth miRNA–gene interactions were not available. Therefore, cancer cohort results were assessed as evidence-recovery outcomes rather than definitive inference-accuracy benchmarks. Across the seven cancer datasets, the full Bayesian network recovered 79.31% of dual-source evidence-supported interactions, compared with 61.89% for the prior-support matched baseline. This 17.42 percentage-point difference indicates that the post-HGT Bayesian topology optimization recovered more previously reported interactions than prior ranking alone within the evaluated candidate space. Because ENCORI/starBase and miRTarBase also contributed to the regulatory prior scaffold, these cancer-cohort values are interpreted as conservative evidence-supported recovery rather than independent experimental validation.

#### 3.1.3. HGT Representation Learning Captures Latent miRNA–Gene Regulatory Structure

**Figure 2:**
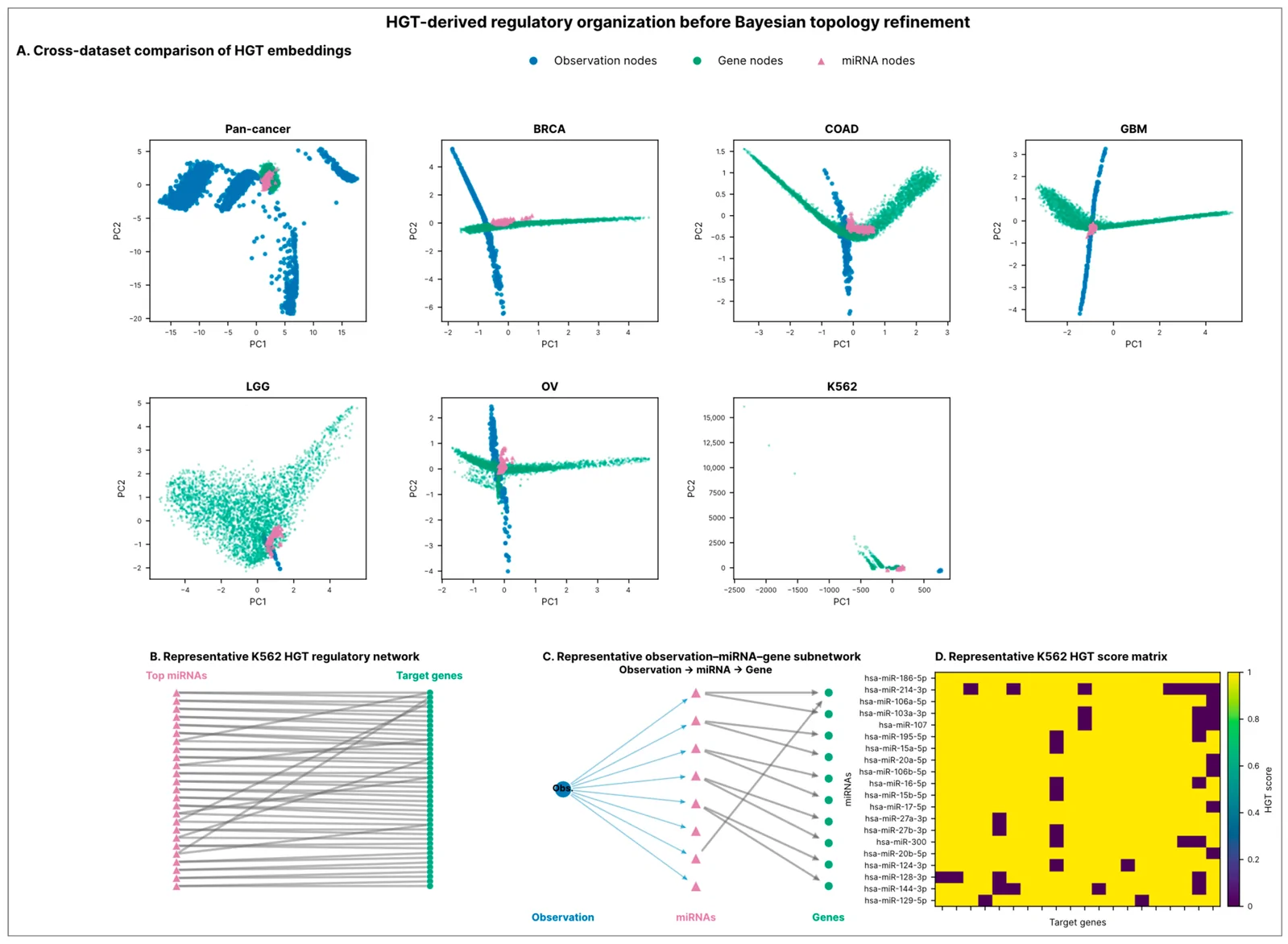
HGT-derived latent representations and regulatory organization prior to Bayesian topology optimization. (**A**) PCA projections of the 128-dimensional HGT embeddings across the seven datasets, illustrating the separation of observation, gene, and miRNA nodes while preserving shared regulatory organization. (**B**) Representative HGT-derived miRNA–gene regulatory network from the K562 single-cell dataset before Bayesian refinement. (**C**) Representative observation–miRNA–gene subnetwork demonstrating expression-supported regulatory relationships within biological contexts. (**D**) Representative HGT regulatory score matrix showing interaction strengths between candidate master regulators and target genes. Similar regulatory structures were observed across all datasets.

HGT representation learning generated 128-dimensional embeddings for genes, miRNAs, and biological observations across all seven datasets, which were projected into two-dimensional PCA space for visualization (Figure 2A). Observation nodes formed dataset-dependent trajectories or clusters, whereas gene and miRNA nodes occupied distinct but connected regions of the latent space. This consistent organization across datasets indicates that relation-aware HGT message passing captured node-type-specific structure while preserving shared regulatory information.

A representative K562 regulatory network further illustrates how the learned embeddings were translated into candidate regulatory interactions before Bayesian refinement (Figure 2B–D). The top HGT-scored miRNA–gene network identified recurrent candidate regulators, while the observation–miRNA–gene subnetwork demonstrated that these interactions were supported by specific biological contexts. The corresponding HGT regulatory score matrix revealed heterogeneous interaction strengths among candidate regulators and their predicted targets. Similar network organization and interaction patterns were observed across the remaining datasets (see more details in Supplementary Materials).

Together, these results support the use of HGT-derived embeddings as an interpretable regulatory representation before Bayesian topology optimization, consistent with heterogeneous graph transformer learning for multi-type graph representation [33].

#### 3.1.4. Bayesian Topology Optimization Refines HGT-Prioritized Regulatory Networks

**Figure 3:**
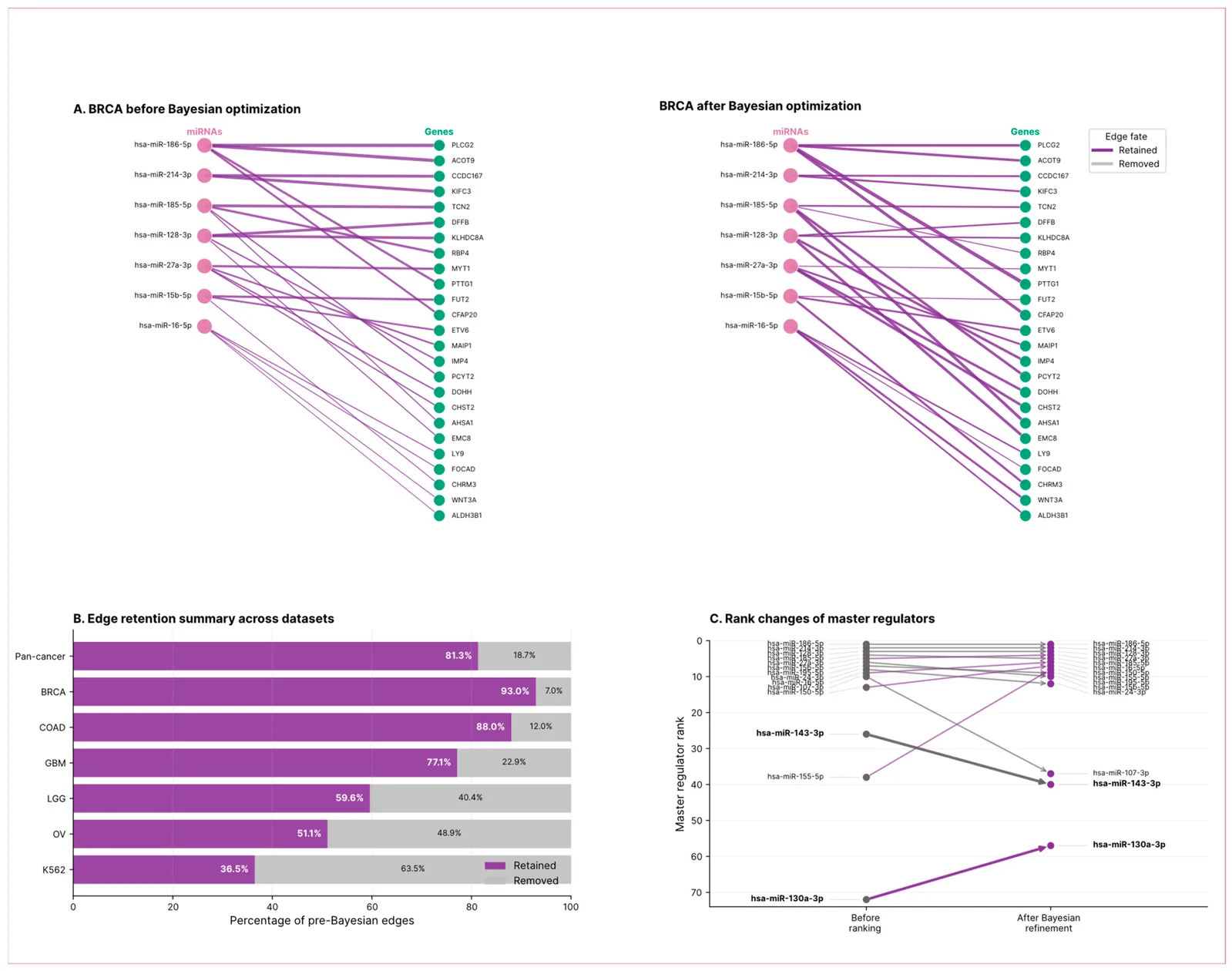
Bayesian topology optimization preserves network structure while refining regulatory specificity. (**A**) Representative BRCA regulatory network before and after Bayesian topology optimization. Purple edges indicate retained regulatory interactions, whereas gray edges indicate interactions removed during refinement. (**B**) Proportion of retained and removed interactions across all datasets, demonstrating that Bayesian optimization preserves the majority of HGT-inferred interactions while eliminating a small subset of low-coherence edges. (**C**) Changes in master regulator rankings following Bayesian refinement, illustrating the transition from globally associated miRNAs to conditionally dominant regulators.

Bayesian topology optimization acted as a selective filter for the HGT-inferred regulatory scaffold, preserving high-confidence candidate interactions while pruning non-informative edges. Figure 3 illustrates the effect of Bayesian topology optimization using the BRCA regulatory network as a representative example. The optimization preserved the overall HGT-derived network architecture while removing a small subset of low-coherence interactions (Figure 3A). Across all datasets, an average of 94.4% of candidate interactions were retained following Bayesian refinement, with approximately 5.6% removed (Figure 3B), demonstrating that the optimization primarily improves network specificity rather than altering global topology. Bayesian refinement also re-prioritized master regulators by emphasizing local conditional dependencies, resulting in substantial rank shifts in several datasets (Figure 3C).

### 3.2. Biological Interpretation of Inferred Regulatory Networks in Cancer

#### 3.2.1. Dominance Share Analysis Identifies Master Regulators and Refined Competition Networks

Dominance Share analysis produced compact, high-precision competition networks that preserved global target-gene coverage while prioritizing the strongest miRNA regulators per gene. Across the seven datasets, these refined networks retained all 25,247 dataset-level target-gene entries, showing that network compression-maintained target-gene representation. The analysis identified distinct dataset-specific master regulators, including miR-27a-3p in the Pan-cancer and LGG cohorts, miR-186-5p in BRCA and COAD, miR-150-5p in GBM, miR-143-3p in OV, and miR-103a-3p in K562 (Table 3). Several regulators displayed broad cross-cohort recurrence, notably miR-186-5p, miR-214-3p, and miR-185-5p, highlighting their conserved role as post-transcriptional hubs in tumorigenesis [53,54]. The refined networks further revealed a deep cooperative regulatory structure. Across all cohorts, we identified 14,048 target genes regulated by three or more miRNAs, and 418 targets subject to co-repression by three or more negatively correlated miRNAs (Table 3). The single-cell K562 cohort exhibited the highest complexity, with 6532 cooperative targets, followed by the BRCA subtype. These findings underscore that the refined networks captured not only individual master regulators but also combinatorial, multi-miRNA regulatory programs, providing a high-resolution mechanistic map of the redundant, buffered control circuits that define tumor-regulatory states [36,37].

#### 3.2.2. Embedding-Derived Context Analysis Reveals Context-Specific miRNA Programs

**Figure 4:**
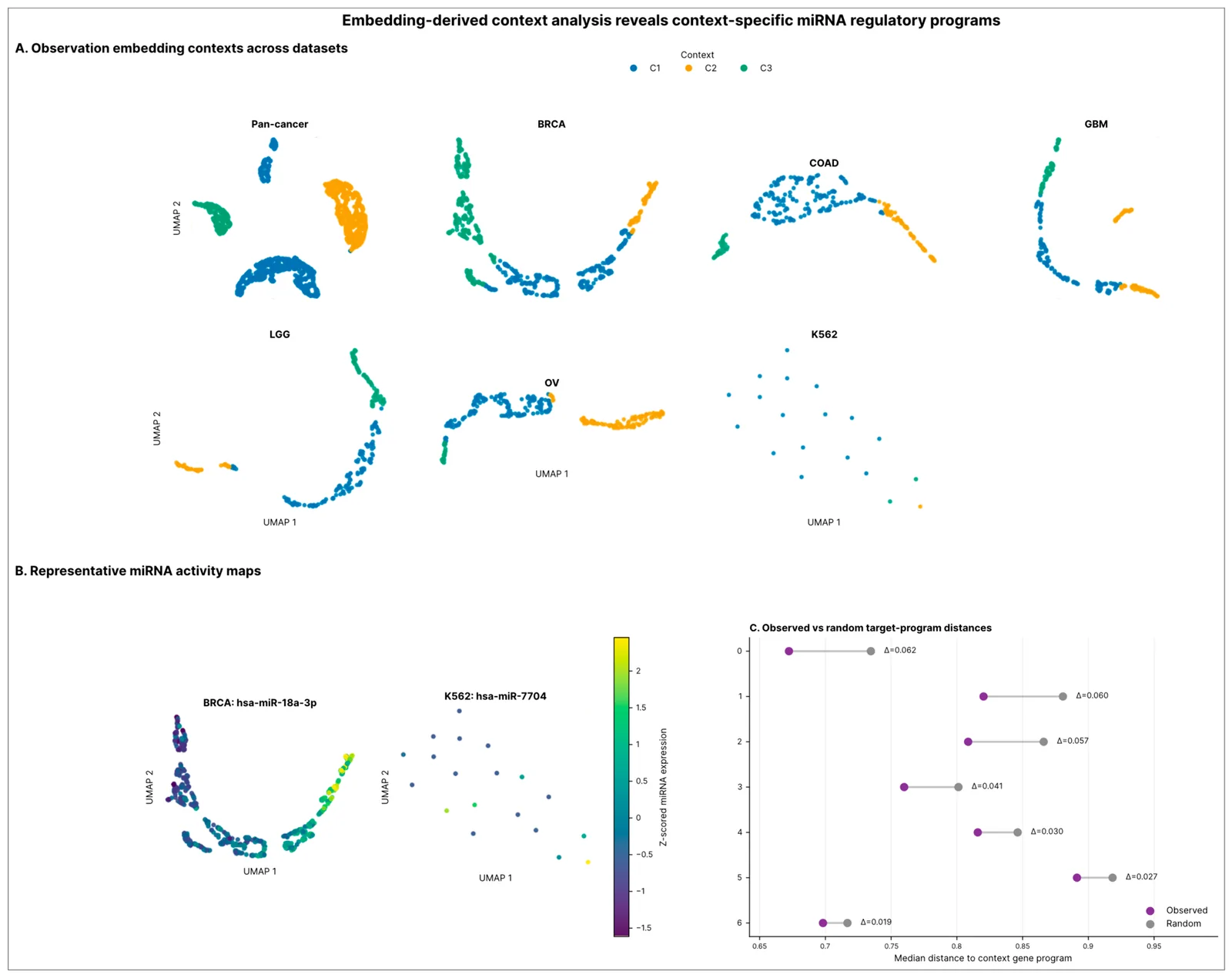
Embedding-derived context analysis reveals context-specific miRNA regulatory programs. (**A**) UMAP projections of HGT observation embeddings across the seven datasets, demonstrating consistent partitioning into three latent biological contexts. (**B**) Representative context-specific activity maps for selected miRNAs in one bulk tumor (BRCA) and one single-cell (K562) dataset, illustrating localized regulatory activity within the learned embedding space. (**C**) Comparison of observed and randomly matched target-program distances across all datasets. Lower observed distances indicate stronger alignment between predicted miRNA target programs and context-specific gene programs. Similar context-dependent activity patterns were observed across all datasets.

Embedding-derived context analysis demonstrated that HGT observation embeddings successfully partitioned each dataset into three distinct context groups (N = 3), indicating that the learned latent space effectively captured structured biological heterogeneity across both bulk tumor and single-cell settings (Figure 4A). Representative activity maps further showed that selected miRNAs exhibited context-specific expression patterns within the learned embedding space (Figure 4B). Similar context-dependent activity was observed across all datasets, including miR-363-3p (Pan-cancer), miR-18a-3p (BRCA), miR-186-3p (COAD), miR-145-3p (GBM), miR-217-3p (LGG), let-7f-3p (OV), and miR-7704 (K562), demonstrating that miRNA regulatory activity is localized to distinct biological contexts rather than uniformly distributed across observations.

Target-program proximity analysis provided an independent computational assessment of the biological coherence of the learned latent space (Figure 4C). Across all seven datasets, predicted miRNA target programs were consistently closer in the embedding space to observed context-specific gene programs than to randomly matched gene sets (permutation test, *p* < 0.05). Median observed program distances were lower than matched random distances in every dataset, with positive random-minus-observed distance shifts ranging from 0.0186 in BRCA to 0.0623 in the Pan-cancer cohort. The largest alignment shifts were observed in the Pan-cancer (0.0623), K562 (0.0604), and OV (0.0575) datasets, indicating particularly strong correspondence between inferred miRNA regulatory programs and context-specific transcriptional states. Collectively, these findings demonstrate that miR-CellMap associates miRNAs with biologically relevant gene programs within distinct cellular or disease contexts, supporting the biological interpretability of the HGT-derived latent representation and its application to heterogeneous regulatory network analysis [33,50].

#### 3.2.3. Cross-Dataset Regulatory Modeling Identifies Recurrent and Cancer-Specific miRNA Regulators

**Figure 5:**
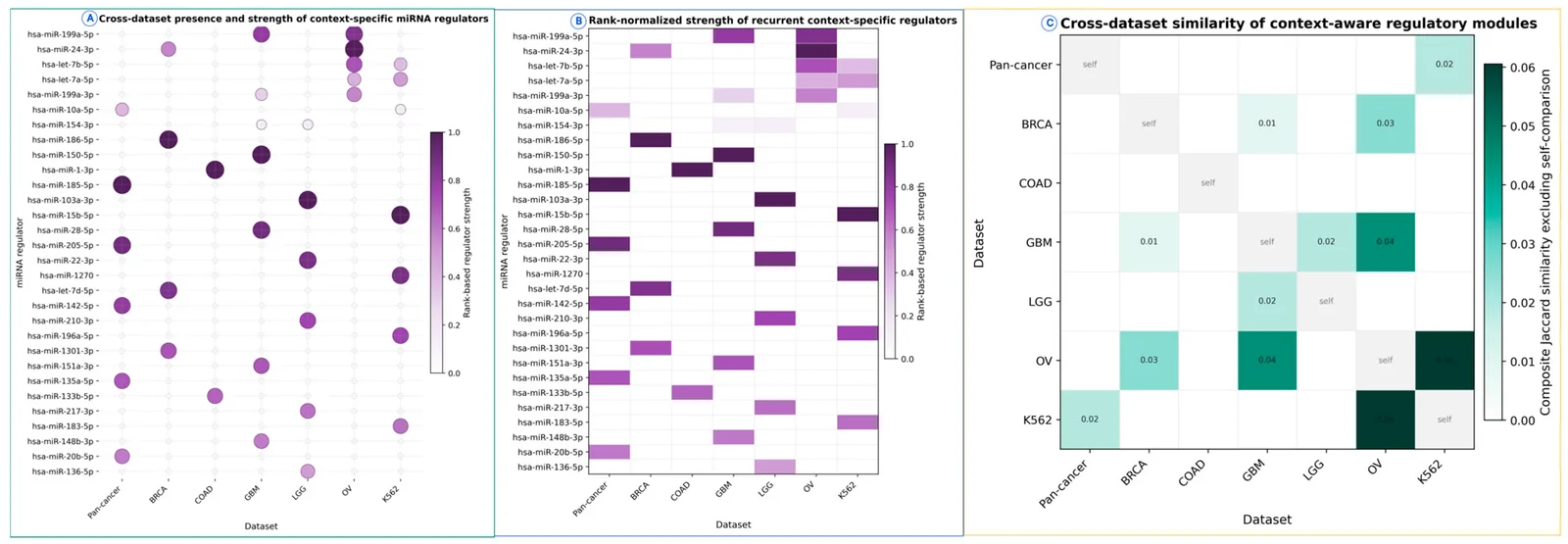
Cross-dataset modeling of recurrent and dataset-specific context-aware miRNA regulators. (**A**) Presence and rank-normalized strength of context-specific miRNA regulators across Pan-cancer, BRCA, COAD, GBM, LGG, OV, and K562 datasets. Larger and darker circles indicate stronger within-dataset regulator ranking, while open circles indicate absence from the displayed top regulator set. (**B**) Rank-normalized heatmap of recurrent and dataset-specific regulator strength. Darker purple indicates stronger regulator rank within a dataset. (**C**) Cross-dataset module similarity heatmap based on composite Jaccard similarity. Diagonal entries are labeled “self” because they represent each dataset compared with itself and are not interpreted as cross-dataset overlap. Off-diagonal values represent similarity between different datasets.

Cross-dataset regulatory modeling compared the top 25 context-specific miRNA regulators across all cohorts, yielding 175 regulator rankings (Figure 5). Among the 30 unique regulators displayed in the cross-dataset matrix, 23 (76.7%) appeared in only one dataset, while 7 (23.3%) appeared in two or more, indicating that the majority of high-ranking miRNA programs are context-specific rather than broadly shared. Recurrent regulators included miR-199a-5p, miR-24-3p, let-7b-5p, let-7a-5p, miR-199a-3p, miR-10a-5p, and miR-154-3p. The OV cohort displayed the highest degree of regulatory overlap, contributing to 5 of the 7 recurrent patterns, particularly with GBM, BRCA, and K562. Conversely, several regulators were uniquely enriched in specific contexts, such as miR-185-5p (Pan-cancer), miR-186-5p (BRCA), miR-1-3p (COAD), miR-150-5p (GBM), miR-103a-3p (LGG), miR-24-3p (OV), and miR-15b-5p (K562).

The module similarity analysis confirmed that context-aware regulatory modules are predominantly dataset-specific. Of the 21 unique cross-dataset comparisons, only 6 showed non-zero module similarity, with off-diagonal Jaccard similarity indices ranging from 0.01 to 0.06. The highest observed overlap occurred between OV and K562 (Jaccard = 0.06), followed by GBM–OV (0.04) and BRCA–OV (0.03). This overlap between OV and K562 indicates limited similarity between the inferred single-cell and bulk regulatory architectures, partially driven by shared *let-7* family regulators. Diagonal entries in the similarity heatmap are labeled “self,” denoting a Jaccard similarity of 1.0 (identity comparison), and are excluded from cross-dataset interpretation. These findings demonstrate that miR-CellMap effectively identifies a conserved core of cancer-regulatory miRNAs while preserving distinct, context-specific regulatory signatures, consistent with the heterogeneous nature of miRNA-mediated post-transcriptional control [10,36,37,54,55].

#### 3.2.4. Context-Aware Regulatory Modules Highlight Dataset-Specific miRNA–Gene Programs

**Figure 6:**
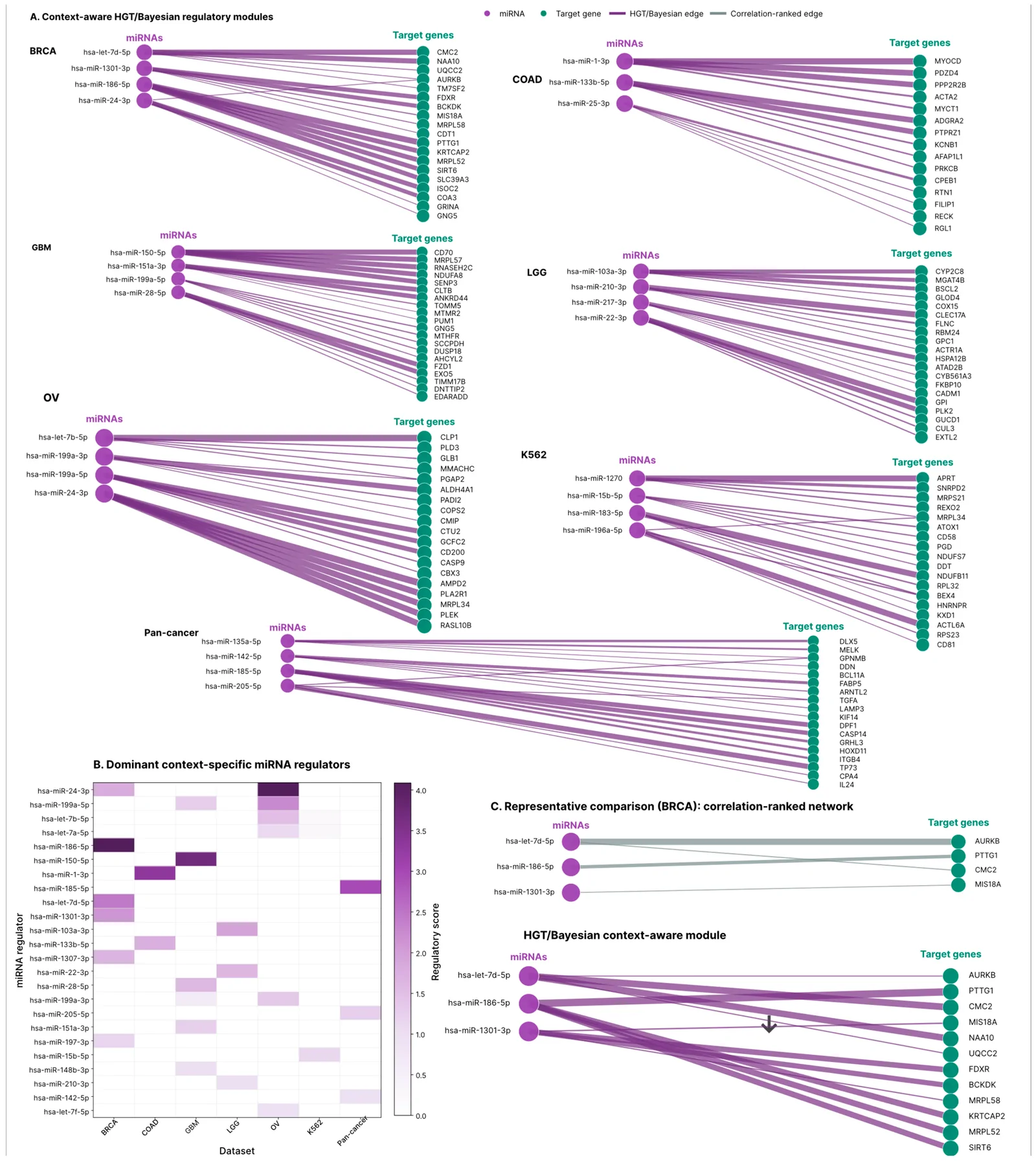
Context-aware miRNA–gene regulatory modules identified across cancer datasets. (**A**) Context-specific HGT/Bayesian regulatory modules extracted from representative latent contexts for each dataset. Purple nodes denote miRNAs, green nodes denote target genes, and edge width reflects the inferred regulatory strength. (**B**) Heatmap summarizing the dominant miRNA regulators and their final regulatory scores across datasets, highlighting both shared and dataset-specific regulatory programs. (**C**) Representative comparison between a correlation-based regulatory network and the corresponding HGT/Bayesian-refined module, illustrating the improved specificity and compact organization achieved through context-aware regulatory inference.

Context-aware regulatory module analysis identified compact miRNA–gene programs within selected HGT-derived contexts across all seven datasets (Figure 6A). Each module comprised three to four dominant miRNA regulators connected to approximately 14–20 high-confidence target genes. Although every dataset exhibited a distinct regulatory composition, OV, GBM, and the Pan-cancer cohort displayed particularly strong lead-regulator dominance, whereas LGG and K562 showed more distributed regulatory control.

Comparison of dominant regulators across datasets further revealed both conserved and context-specific regulatory programs (Figure 6B). While several miRNAs appeared as leading regulators in multiple datasets, the dominant regulatory architecture remained highly dataset dependent, indicating that miR-CellMap captures both shared and disease-specific patterns of miRNA regulation. These differences suggest that context-aware network inference identifies regulatory hierarchies that are tailored to the biological state represented by each latent context rather than relying on globally shared associations.

A representative comparison between correlation-based and HGT/Bayesian-derived regulatory modules illustrates the increased specificity achieved through Bayesian refinement (Figure 6C). Correlation-based networks produced diffuse association patterns with numerous weakly connected interactions, whereas miR-CellMap recovered compact regulatory hubs centered on dominant miRNA regulators and their highest-confidence target genes. Across all datasets, the refined modules exhibited clearer regulator–target organization and more interpretable edge-strength distributions, supporting a context-aware view of combinatorial miRNA regulation in cancer [36,37,50,53].

#### 3.2.5. Literature-Based Functional Annotation of Top Regulators and Multi-miRNA Target Modules

Literature-based functional validation of the top-ranked regulators showed that most predicted master miRNAs were linked to cancer-relevant regulatory processes. Across the seven datasets, 66 of 70 top-ranked regulators (94.3%) had literature-supported cancer pathway associations. These included cell-cycle and apoptosis-associated regulators such as miR-186-5p, miR-16-5p, miR-195-5p, and miR-25-3p; PI3K/Akt- and EMT-associated regulators such as miR-214-3p and miR-130a-3p; TGF-β- and epithelial plasticity-associated regulators such as miR-27a-3p; and RAS/HMGA2-associated let-7 family regulators [36,37,53,55]. The strongest lead regulators were also supported by large target programs and high final regulatory scores (Table 4). miR-103a-3p in K562 had the largest target set, with 604 targets and a final HGT/Bayesian score sum of 109.66. In the bulk tumor datasets, strong lead regulators included miR-27a-3p in LGG with 249 targets, miR-186-5p in COAD with 247 targets, miR-186-5p in BRCA with 222 targets, miR-150-5p in GBM with 156 targets, miR-143-3p in OV with 150 targets, and miR-27a-3p in Pan-cancer with 92 targets. Across all top statistical regulators, target counts ranged from 29 to 604, final regulatory score sums ranged from 10.21 to 109.66, and high-coherence edge fractions reached up to 46.74%. Together, these findings indicate that the strongest miR-CellMap regulators are not only statistically prioritized but also enriched for known cancer-related regulatory functions, supporting the biological relevance of the inferred regulatory networks [10,54].

## 4. Discussion

This study developed and evaluated miR-CellMap, a context-aware graph transformer framework for miRNA–gene regulatory inference across matched single-cell and bulk cancer datasets. The working hypothesis was that integrating paired miRNA/mRNA expression, curated regulatory priors, heterogeneous graph representation learning, Bayesian topology optimization, and Dominance Share-based competition analysis would produce more interpretable and biologically meaningful miRNA–gene regulatory networks than expression association alone. The results support the utility of the integrated strategy. Across the seven cancer datasets, the full Bayesian miR-CellMap network recovered 79.31% of dual-source evidence-supported interactions, compared with 61.89% for the prior-support matched baseline; this result was interpreted as conservative evidence recovery rather than independent inference accuracy. These findings suggest that combining graph-based regulatory representation with probabilistic topology refinement can improve recovery of evidence-supported miRNA–gene interactions and address key limitations of correlation-based or prior-only approaches. This is consistent with previous studies showing that miRNA regulation is combinatorial, context-dependent, and difficult to resolve from sequence prediction or expression association alone [36,37].

A key contribution of miR-CellMap is its explicit representation of biological context. In the single-cell dataset, context corresponded to matched K562 cell profiles, whereas in bulk tumor datasets, context represented tumor samples or subtype-defined molecular profiles. HGT representation learning separated observations into latent context groups and generated embeddings for observations, miRNAs, and genes within a shared regulatory space. The embedding-derived context analysis further showed that predicted miRNA target programs were closer to observed context gene programs than random matched programs in all seven datasets, with positive random-minus-observed shifts ranging from 0.0186 to 0.0623. This indicates that the learned embeddings captured context-associated structure beyond that observed for the matched random gene programs. These findings are consistent with the broader view that cellular state, tumor heterogeneity, and multi-omic regulatory activity are shaped by latent biological context [22,23,29,33].

Bayesian topology optimization and Dominance Share analysis improved the interpretability of the inferred networks by converting dense candidate interactions into refined competition networks. The refined networks preserved all 25,247 dataset-level target-gene entries while prioritizing a smaller set of competing miRNA regulators per gene. This is biologically important because miRNA regulation is not usually one-to-one; multiple miRNAs can converge on the same target, and individual miRNAs can regulate multiple genes within related pathways [36,37]. The identification of 14,048 cooperative targets and 418 co-repressed targets supports this combinatorial regulatory model. Several regulators prioritized by miR-CellMap, including miR-186-5p, miR-214-3p, miR-27a-3p, miR-24-3p, miR-101-3p, miR-143-3p, let-7 family members, and miR-103a-3p, have been previously associated with cancer-related processes such as cell-cycle control, apoptosis, PI3K/Akt signaling, epithelial plasticity, KRAS/MAPK signaling, and tumor suppressive regulation [10,53,54,55].

The cross-dataset analysis showed that miRNA regulatory programs were largely context-specific, with a smaller subset of recurrent regulators shared across datasets. Among the 30 regulators displayed in the cross-dataset matrix, 23 appeared in only one dataset, whereas 7 appeared in two datasets. This pattern is biologically plausible because miRNA activity depends on tumor lineage, target availability, expression context, and regulatory state. Module similarity analysis also showed low cross-dataset overlap, with off-diagonal composite Jaccard similarities ranging from 0.01 to 0.06. The strongest overlap was observed between OV and K562, suggesting a limited but detectable connection between a bulk ovarian cancer regulatory program and a single-cell leukemia-derived regulatory context. Although this overlap should not be interpreted as disease equivalence, it may reflect shared regulatory mechanisms captured across biological resolutions, particularly through recurrent let-7 family signals. This finding highlights a potential bridge between bulk-level tumor analyses and higher-resolution cellular regulatory mechanisms.

The literature-based functional annotation further supports the biological plausibility of the inferred networks. Across the top-ranked regulators, 66 of 70 miRNAs had literature-supported cancer pathway associations, including pathways related to TGF-β signaling, PI3K/Akt signaling, MAPK/KRAS signaling, hypoxia response, immune-related cancer signaling, apoptosis, and cell-cycle regulation. Lead regulators such as miR-27a-3p in Pan-cancer and LGG, miR-186-5p in BRCA and COAD, miR-150-5p in GBM, miR-143-3p in OV, and miR-103a-3p in K562 were supported by both network-level ranking and cancer-relevant biological annotation. These findings suggest that miR-CellMap prioritizes regulators with plausible biological relevance, rather than producing only statistically high-scoring associations.

Several limitations should be considered. First, most datasets analyzed in this study were bulk tumor cohorts, where biological context reflects sample-level tumor heterogeneity rather than direct single-cell states. Although miR-CellMap was also evaluated on matched single-cell K562 co-profiling data, this dataset contained only 19 matched profiles, limiting generalization of single-cell-specific conclusions. Second, curated regulatory priors from resources such as miRTarBase and ENCORI/starBase are incomplete and may be biased toward well-studied miRNAs and cancer genes [15,16,17]. Third, the high-confidence negative evaluation setting improves interpretability by excluding prior-supported non-reference interactions from the negative class, but it should not be interpreted as experimental confirmation of all predicted interactions. Finally, the functional validation presented here is based on computational evidence and literature-supported pathway annotation; experimental perturbation studies are needed to confirm causal miRNA–target relationships.

Future work should extend miR-CellMap to larger matched single-cell miRNA/mRNA co-profiling datasets, spatial multi-omic data, perturbation-based datasets, and clinical outcome-linked cohorts. Integrating survival, treatment response, mutation status, and tumor microenvironment annotations could help connect inferred miRNA regulatory programs to clinically relevant cancer phenotypes. Experimental validation using miRNA knockdown, overexpression, CRISPR-based perturbation, or reporter assays would also help test high-priority regulators and cooperative target modules. Overall, miR-CellMap provides an interpretable computational framework for mapping context-specific miRNA–gene regulatory landscapes across cancer datasets and offers a foundation for future studies of non-coding RNA regulation in precision oncology.

## 5. Conclusions

This study presents miR-CellMap, a context-aware graph transformer framework for inferring miRNA–gene regulatory networks across matched single-cell and bulk cancer datasets. By integrating paired miRNA/mRNA expression profiles, curated regulatory priors, heterogeneous graph transformer learning, Bayesian topology optimization, and Dominance Share-based competition analysis, miR-CellMap recovered evidence-supported miRNA–gene interactions and identified interpretable regulatory programs across seven datasets. The full Bayesian network achieved strong benchmark performance under the high-confidence negative setting, while the refined competition networks preserved target-gene coverage and highlighted recurrent and dataset-specific master regulators.

Across bulk tumor and single-cell contexts, miR-CellMap identified biologically relevant miRNAs, including miR-186-5p, miR-214-3p, miR-27a-3p, miR-24-3p, miR-101-3p, miR-143-3p, let-7 family members, and miR-103a-3p. The framework also revealed cooperative and co-repressed target modules, embedding-derived context-specific miRNA programs, and mostly dataset-specific regulatory architectures with limited recurrent cross-dataset overlap. Together, these findings support miR-CellMap as an interpretable computational strategy for mapping context-dependent miRNA regulatory landscapes offering a scalable foundation for prioritizing therapeutic targets and deciphering non-coding regulatory circuits in precision oncology, and provide a foundation for future experimental and clinical studies of non-coding RNA regulation.

## Figures and Tables

**Table 1:** Descriptive statistics of the heterogeneous graphs constructed for miR-CellMap across bulk tumor and single-cell datasets. Context nodes correspond to individual tumor samples in bulk datasets or matched single cells in the K562 co-profiled dataset. Gene and miRNA nodes represent retained molecular features after preprocessing and feature selection. Prior-supported interactions denote candidate miRNA–gene regulatory interactions retained after dataset-specific filtering of the integrated regulatory prior. Bayesian-optimized interactions represent the final regulatory interactions retained after Bayesian topology optimization and network refinement.

Dataset	Biological Contexts	Gene Nodes	miRNA Nodes	Prior-Supported Interactions	Bayesian-Optimized Interactions
Pan-cancer	3378	3217	383	15,271	12,361
BRCA	671	5000	352	58,543	54,015
COAD	260	5000	376	34,774	30,329
GBM	244	5000	340	25,818	19,815
LGG	247	5000	340	21,980	13,010
OV	284	5000	336	20,417	10,369
K562	19	23,284	2822	275,658	99,506

**Table 2:** Ground-truth benchmark and discovery-oriented evidence-recovery summary. The A549 setting represents benchmark-style evaluation because a verified interaction set was available. The cancer cohort setting was treated as discovery-oriented evidence recovery because no independent held-out gold-standard miRNA–gene interaction set was available. Cancer recovery values summarize the proportion of dual-source evidence-supported interactions recovered by the inferred networks and should not be interpreted as definitive classification accuracy.

Assessment Setting	Datasets	Reference Evidence	Main Result	Interpretation
Ground-truth benchmark	A549 single-cell benchmark	Experimentally validated miRNA–gene interactions	220/255 recovered; sensitivity = 86.27%; F1 = 92.63%; MCC > 0.928	Independent benchmark against experimentally validated interactions
Discovery-oriented evidence recovery	Seven cancer datasets	Dual-source literature-supported interactions	Bayesian recovery = 79.31%; prior-support baseline = 61.89%	Evidence recovery only; not an independent inference-accuracy benchmark

**Table 3:** Dominance Share-based master regulators and cooperative target modules across datasets. The top refined master regulators are the three highest-ranked miRNAs identified by Dominance Share analysis in each dataset. Target gene coverage reports the number of unique target genes represented in the refined regulatory network relative to the corresponding Bayesian-optimized network. Cooperative targets are genes regulated by at least three miRNAs, whereas co-repressed targets are cooperative targets associated with at least three negatively correlated miRNA regulators.

Dataset	Top Refined Master Regulators	Target Genes	Cooperative Targets	Co-Repressed Targets
Pan-cancer	miR-27a-3p; miR-185-5p; miR-214-3p	1459	612	8
BRCA	miR-186-5p; miR-155-5p; miR-214-3p	2855	2002	46
COAD	miR-186-5p; miR-128-3p; miR-106b-5p	2911	1840	32
GBM	miR-150-5p; miR-181b-5p; miR-29b-3p	2389	1372	19
LGG	miR-27a-3p; miR-22-3p; miR-214-3p	2345	904	13
OV	miR-143-3p; miR-101-3p; miR-195-5p	2325	786	14
K562	miR-103a-3p; miR-101-3p; let-7a-5p	10,963	6532	286

**Table 4:** Functional support for lead miRNA master regulators across datasets. Lead master regulators represent the highest-ranked miRNA in each dataset based on the refined master regulator table. Target count indicates the number of genes associated with each lead regulator. The final HGT/Bayesian score sum summarizes the total regulatory strength assigned to each lead regulator across its inferred target program.

Dataset	Lead Master Regulator	Target Count	HGT/Bayesian Score Sum	Key Supported Cancer-Related Pathway
Pan-cancer	miR-27a-3p	92	23.55	TGF-β signaling; proliferation; epithelial plasticity
BRCA	miR-186-5p	222	27.75	Cell-cycle and apoptosis-associated cancer regulation
COAD	miR-186-5p	247	36.12	Cell-cycle and apoptosis-associated cancer regulation
GBM	miR-150-5p	156	22.27	Immune/cancer signaling; hematopoietic and tumor-associated regulation
LGG	miR-27a-3p	249	67.23	TGF-β signaling; proliferation; epithelial plasticity
OV	miR-143-3p	150	55.10	KRAS/MAPK-associated regulation; tumor suppressive signaling
K562	miR-103a-3p	604	109.66	Metabolic and oncogenic signaling; epithelial plasticity

## Data Availability

The datasets analyzed in this study are publicly available from their original sources. The matched single-cell miRNA/mRNA co-profiling dataset is available from the Gene Expression Omnibus under accession GSE114071. Bulk cancer miRNA and mRNA datasets were obtained from the MLOmics resource, and the download link is provided in the project repository README. Curated miRNA–gene regulatory evidence was obtained from miRTarBase and ENCORI/starBase. No new raw sequencing data were generated in this study. The computational framework used in this study is based on the miR-CellMap pipeline, which is available at https://github.com/sbbi-unl/miR-CellMap (accessed on 6 January 2026). The present cancer-focused analysis was implemented using the same core workflow with dataset-specific preprocessing, graph construction, Bayesian topology optimization, and downstream regulatory interpretation steps. Large public datasets are not redistributed in the repository; instead, access links and preprocessing instructions are provided in the repository README.

## Supplementary Materials

The following supporting information can be downloaded at: https://www.mdpi.com/article/10.3390/genes17080846/s1, Supplementary Figure S1. HGT-derived regulatory organization before Bayesian topology refinement across bulk tumor and single-cell datasets. Supplementary Figure S2. Complete Bayesian topology optimization results across all datasets. Supplementary Figure S3. Complete embedding-derived context analyses for all datasets. Supplementary Figure S4. Complete context-aware regulatory modules across all datasets.

## Abbreviations

The following abbreviations are used in this manuscript:

BN	Bayesian Network
BRCA	Breast Invasive Carcinoma
CC	Correlation Coefficient
COAD	Colon Adenocarcinoma
CRISPR	Clustered Regularly Interspaced Short Palindromic Repeats
EMT	Epithelial–Mesenchymal Transition
ENCORI	Encyclopedia of RNA Interactomes
GBM	Glioblastoma Multiforme
GRN	Gene Regulatory Network
GS	Gold-Standard cancer subtype cohort
HGT	Heterogeneous Graph Transformer
HMGA2	High Mobility Group AT-hook 2
K562	Human Chronic Myelogenous Leukemia Cell Line
KRAS	Kirsten Rat Sarcoma Viral Oncogene Homolog
LGG	Brain Lower Grade Glioma
MAPK	Mitogen-Activated Protein Kinase
MCC	Matthews Correlation Coefficient
miRNA	microRNA
miRTarBase	microRNA Target Interaction Database
mRNA	Messenger RNA
ncRNA	Non-coding RNA
OV	Ovarian Serous Cystadenocarcinoma
PI3K/Akt	Phosphoinositide 3-Kinase/Protein Kinase B
TGF-β	Transforming Growth Factor Beta
UMAP	Uniform Manifold Approximation and Projection
