# Supplementary material for: A Context-Aware Graph Transformer Framework for microRNA–Gene Regulatory Inference Across Bulk Tumor and Single-Cell Cancer Data
Source: Genes (Basel). 2026 Jul 23;17(8):846. doi: 10.3390/genes17080846 (PMC13512291; doi:10.3390/genes17080846)
Supplement: Supplementary file 1 [file genes-17-00846-s001.zip › genes-4407711-supplementary.pdf]

Supplementary Materials

This supplementary document provides expanded versions of the main-text figures together with complete dataset-specific visualizations that support the analyses presented in the manuscript. These figures include detailed results for all datasets, whereas representative examples are shown in the main text for clarity.

Supplementary Figure S1. HGT-derived regulatory organization before Bayesian topology refinement across bulk tumor and single-cell datasets.
Panels show (a) GS-BRCA, (b) GS-COAD, (c) GS-GBM, (d) GS-LGG, (e) GS-OV, (f) GSE114071_K562, and (g) MLOmics selected pan-cancer. Each dataset includes four components: **(A)** PCA projection of the 128-dimensional HGT embeddings for observation, gene, and miRNA nodes; **(B)** top HGT-scored miRNA–gene regulatory network prior to Bayesian topology refinement; **(C)** representative observation–miRNA–gene regulatory subnetwork; and **(D)** HGT regulatory score matrix for the highest-ranked miRNA–gene interactions. These expanded visualizations complement the representative examples shown in **Figure 2** of the main text.

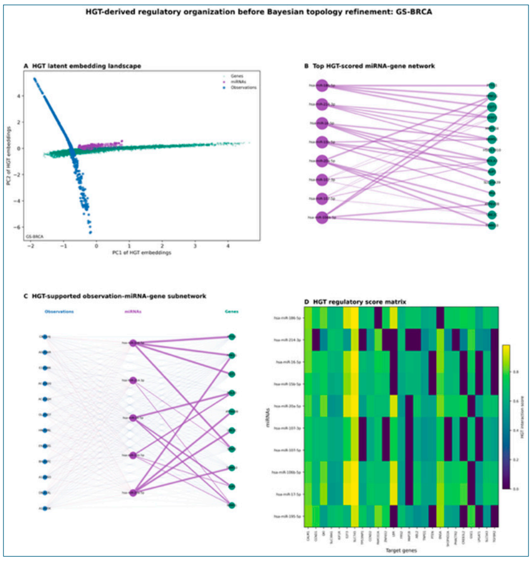

(**a**) GS-BRCA HGT-derived regulatory organization before Bayesian topology refinement

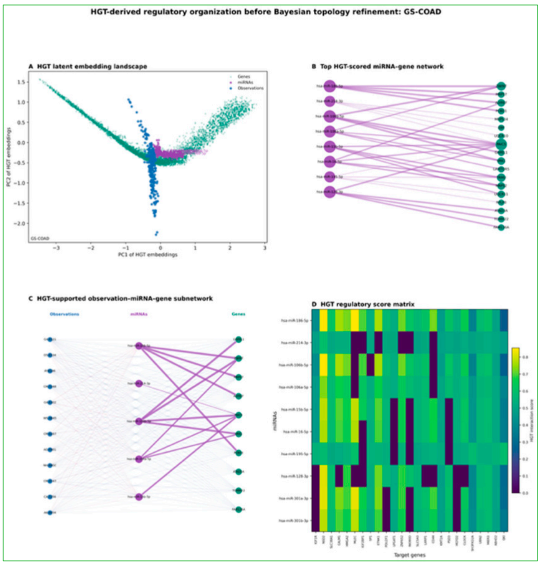

(**b**) GS-COAD HGT-derived regulatory organization before Bayesian topology refinement

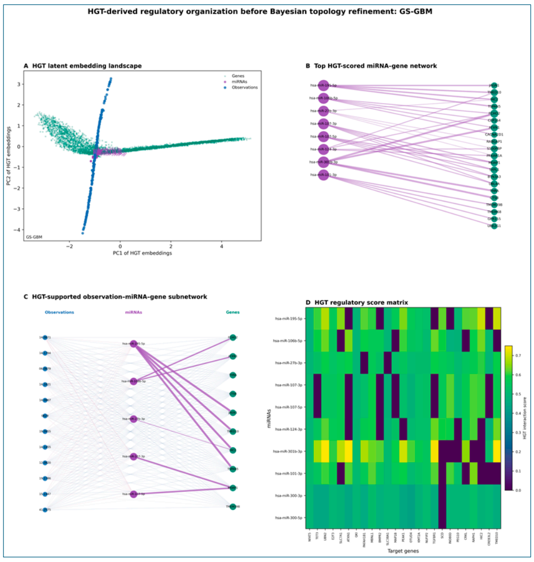

(c) GS-GBM HGT-derived regulatory organization before Bayesian topology refinement

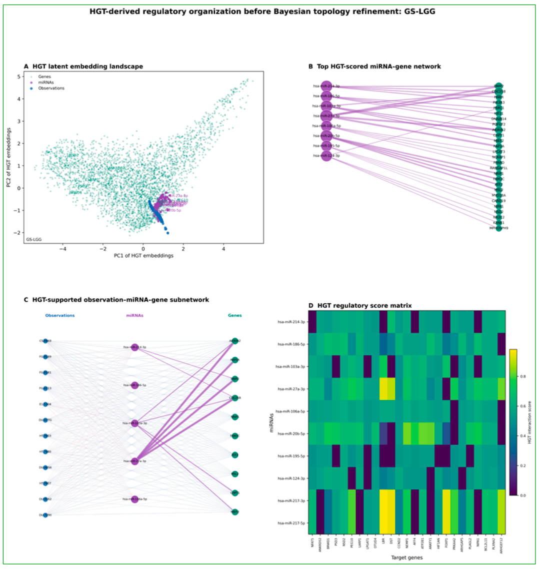

(d) GS-LGG HGT-derived regulatory organization before Bayesian topology refinement

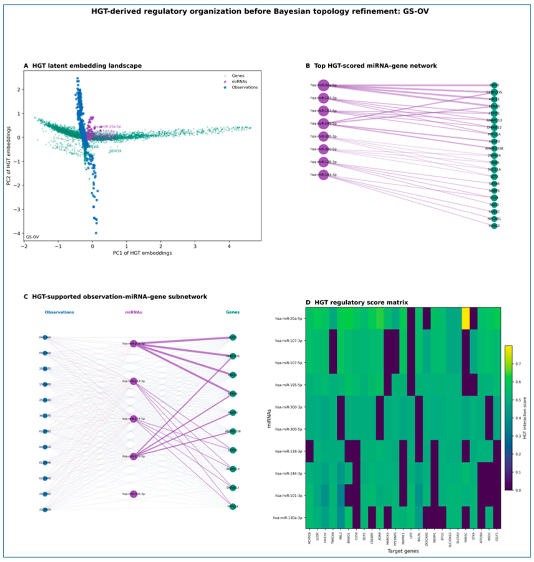

(e) GS-OV HGT-derived regulatory organization before Bayesian topology refinement

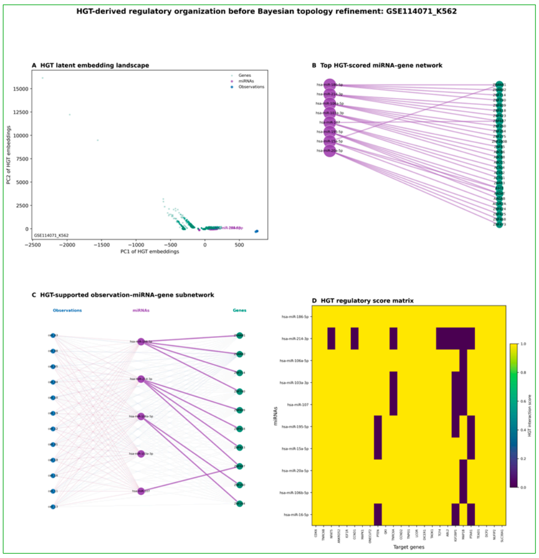

(f) GSE114071_K562 HGT-derived regulatory organization before Bayesian topology refinement

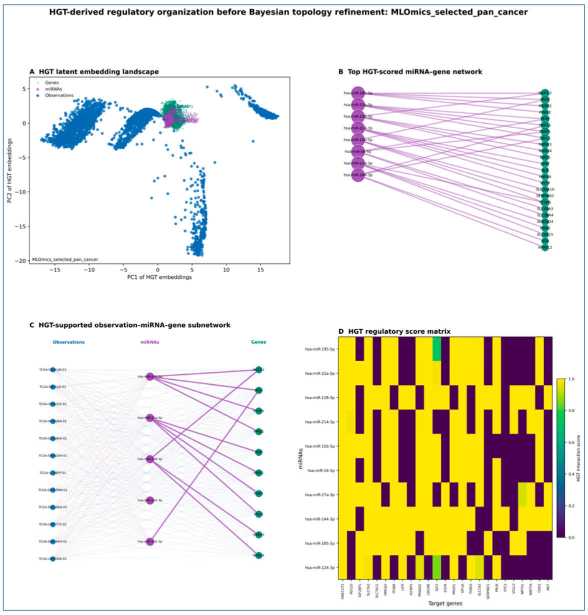

(g) MLOmics selected pan-cancer HGT-derived regulatory organization before Bayesian topology refinement

Supplementary Figure S2. Complete Bayesian topology optimization results across all datasets (expanded version of Figure 3).

Pre- and post-Bayesian regulatory network comparison across bulk tumor and single-cell datasets. Panels (a–g) represent the following cohorts: (a) GS-BRCA, (b) GS-COAD, (c) GS-GBM, (d) GS-LGG, (e) GS-OV, (f) GSE114071 (K562), and (g) Pan-cancer. Within each dataset subfigure: (A) pre-Bayesian regulatory network inferred from HGT-attention scores; (B) Bayesian-optimized regulatory network following topological refinement; (C) edge fate mapping, where purple edges indicate retained regulatory interactions and grey dashed edges indicate removed candidate interactions; and (D) master regulator rank shifts, demonstrating the re-prioritization of miRNA regulatory dominance before and after optimization.

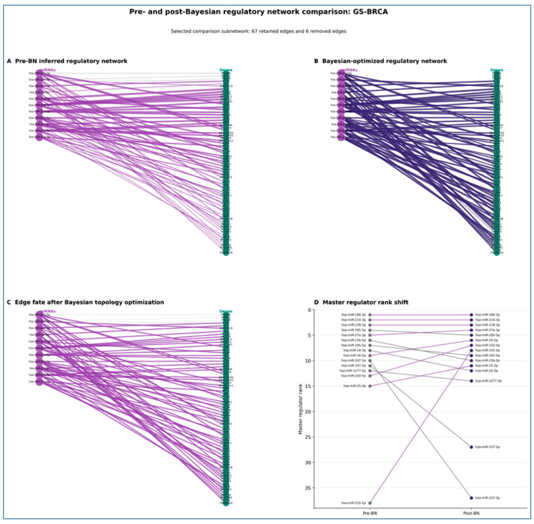

(**a**) GS-BRCA pre and post Bayesian regulatory network comparison

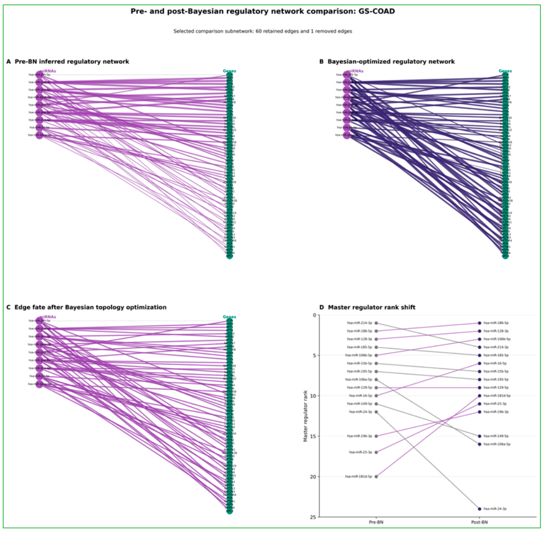

(**b**) GS-COAD pre and post Bayesian regulatory network comparison

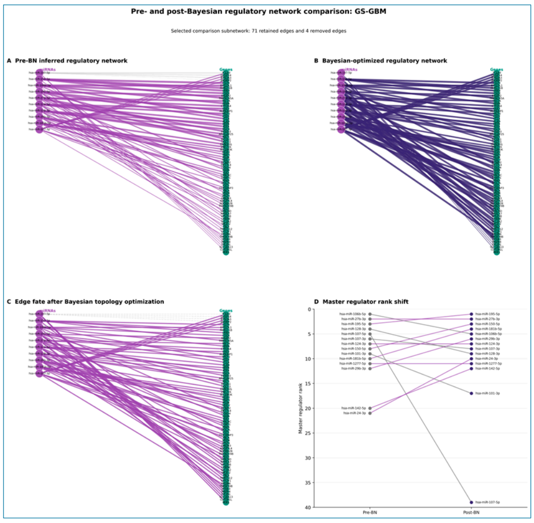

(c) GS-GBM pre and post Bayesian regulatory network comparison

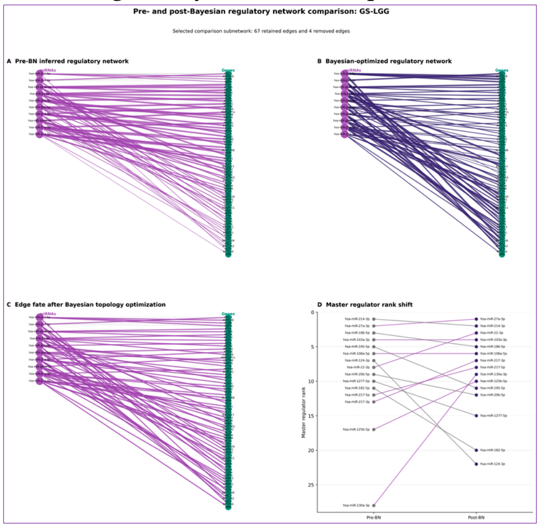

(d) GS-LGG pre and post Bayesian regulatory network comparison

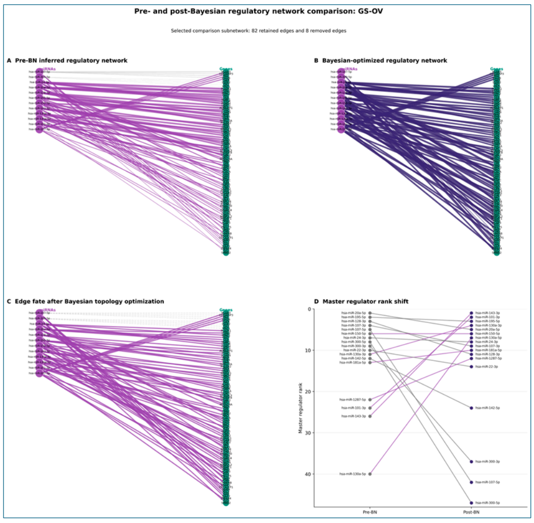

(e) GS-OV pre and post Bayesian regulatory network comparison

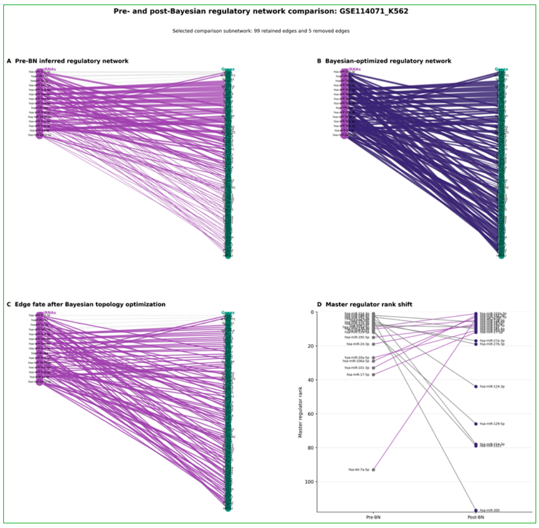

(f) GSE114071_K562 pre and post Bayesian regulatory network comparison

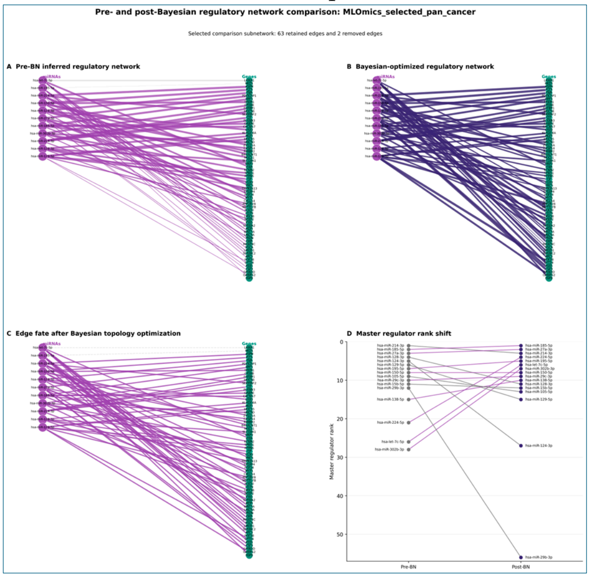

(g) MLOmics selected pan-cancer pre and post Bayesian regulatory network comparison

While certain high-impact regulators exhibited rank stability, such as miR-186-5p and miR-214-3p in GS-BRCA, other datasets underwent significant rank reorganization. For example, in GS-OV, GS-LGG, and the K562 single-cell cohort, miRNAs previously ranked low (e.g., miR-143-3p in GS-OV and miR-130a-3p in GS-LGG) were elevated to top-tier status following the Bayesian refinement. These rank shifts suggest that HGT-derived scores may be influenced by global graph topology, whereas Bayesian refinement emphasizes local conditional dependencies. Conversely, the Bayesian step re-weights these regulators based on their local conditional dependency with specific gene targets. Consequently, the final networks reflect a more granular, dataset-specific hierarchy of miRNA control, shifting the prioritization from broadly associated miRNAs toward locally supported candidate regulators. focus from "statistically associated" miRNAs to "conditionally dominant" regulatory drivers.

Supplementary Figure S3. Complete embedding-derived context analyses for all datasets (expanded version of Figure 4).

HGT embedding-derived observation context analysis across single-cell and bulk cancer datasets. (left) First composite panel showing 1, GSE114071_K562; 2, GS-OV; and 3, GS-COAD. (right) Second composite panel showing 4, GS-GBM; 5, GS-BRCA; 6, GS-LGG; and 7, MLOmics selected pan-cancer. Within each dataset-specific row, A shows observation embedding contexts projected by UMAP, B shows the context activity pattern of a selected miRNA using z-scored expression, and C compares the proximity of observed miRNA target programs with random matched gene programs. Dashed vertical lines indicate median program distance values for observed and random matched programs. Lower observed distances indicate stronger proximity between predicted miRNA target programs and context-specific gene programs.

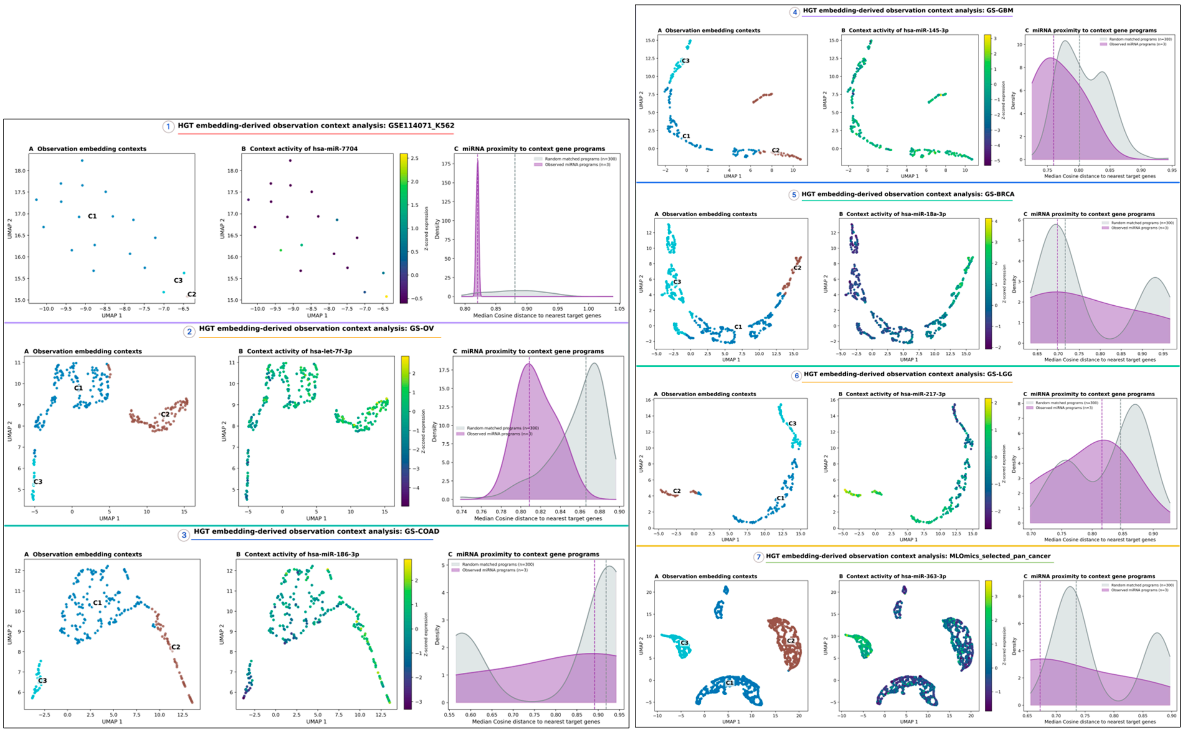

Supplementary Figure S4. Complete context-aware regulatory modules across all datasets (expanded version of Figure 6).

Context-aware miRNA–gene regulatory modules across cancer datasets. (left) First composite panel showing GS-GBM, GSE114071_K562, and GS-COAD. (right) Second composite panel showing GS-BRCA, GS-LGG, MLOmics selected pan-cancer, and GS-OV. Within each dataset row, subpanel A shows the context-aware HGT/Bayesian miRNA–gene regulatory module for the selected embedding-derived context, subpanel B shows the context-specific regulator ranking by final regulatory score, and subpanel C shows the correlation-ranked comparison network for the same selected context. Purple nodes represent miRNAs, green nodes represent target genes, purple edges represent final HGT/Bayesian regulatory edges, and grey edges represent correlation-ranked edges.

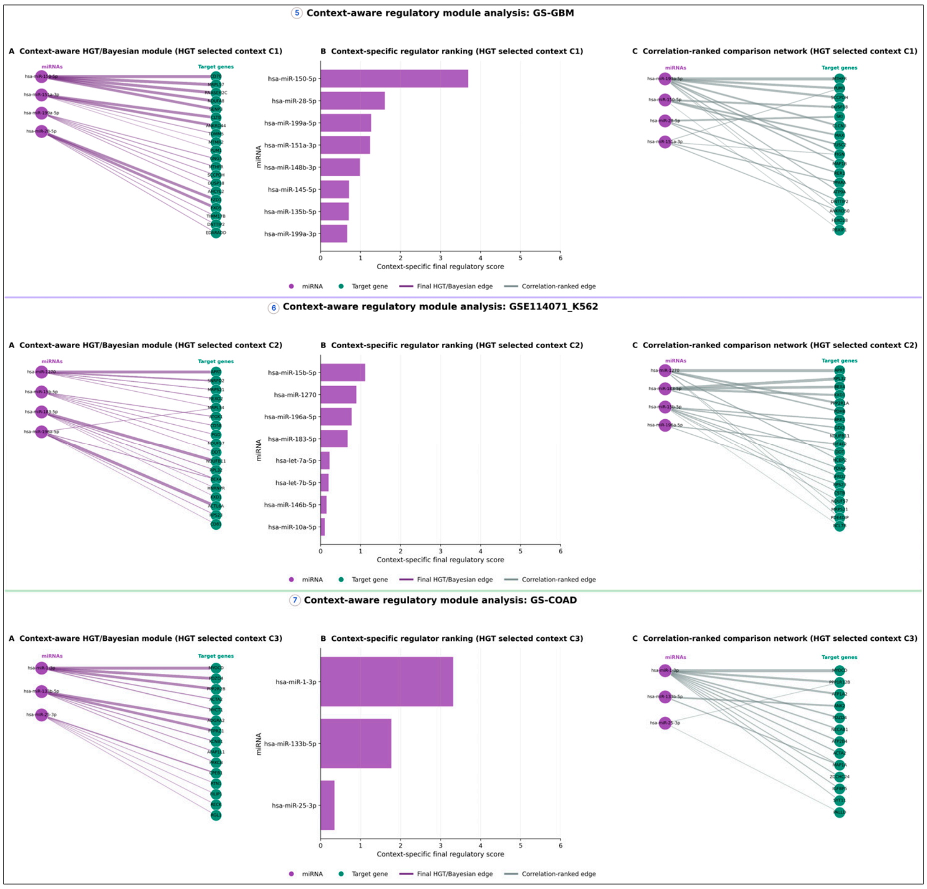

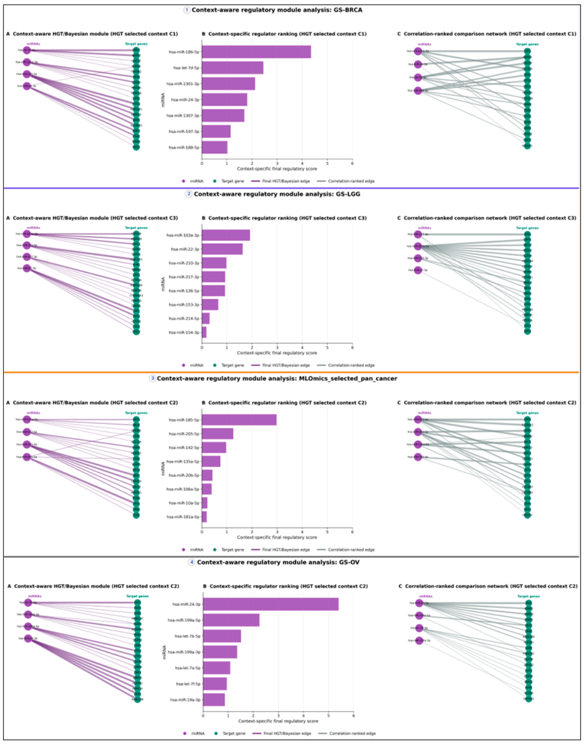

The strongest context-specific signal was observed in OV, where miR-24-3p emerged as the dominant regulator with a final regulatory score of approximately 5.4. Other leading regulators included miR-186-5p in BRCA, approximately 4.4; miR-150-5p in GBM, approximately 3.7; miR-1-3p in COAD, approximately 3.3; miR-185-5p in Pan-cancer, approximately 3.0; miR-103a-3p in LGG, approximately 1.9; and miR-15b-5p in K562, approximately 1.1. OV, GBM, and Pan-cancer showed clear lead-regulator dominance, whereas LGG and K562 displayed more distributed regulatory contributions.
